# A secreted form of chorismate mutase (Rv1885c) in *Mycobacterium bovis* BCG contributes to pathogenesis by inhibiting mitochondria-mediated apoptotic cell death of macrophages

**DOI:** 10.1186/s12929-023-00988-2

**Published:** 2023-12-18

**Authors:** Mi-Hyun Lee, Hye Lin Kim, Hyejun Seo, Sangkwon Jung, Bum-Joon Kim

**Affiliations:** 1 Department of Microbiology and Immunology, College of Medicine, Seoul National University, 103 Daehak-Ro, Jongno-Gu, Seoul, 03080 Republic of Korea; 2 Department of Biomedical Sciences, College of Medicine, Seoul National University, Seoul, 03080 Republic of Korea; 3 BK21 FOUR Biomedical Science Project, Seoul National University College of Medicine, Seoul, 03080 Republic of Korea; 4 Liver Research Institute, College of Medicine, Seoul National University, Seoul, 03080 Republic of Korea; 5 Cancer Research Institute, College of Medicine, Seoul National University, Seoul, 03080 Republic of Korea; 6 Seoul National University Medical Research Center (SNUMRC), Seoul, 03080 Republic of Korea

**Keywords:** *M. bovis* BCG, *M. tuberculosis*, *Mycobacterium tuberculosis* chorismate mutase (TBCM), Rv1885c, Mitochondrial dysfunction, Intrinsic apoptosis, Macrophages, Deletion mutant, Virulence factor

## Abstract

**Background:**

*Mycobacterium tuberculosis* is the causative agent of tuberculosis (TB), and its pathogenicity is associated with its ability to evade the host defense system. The secretory form of the chorismate mutase of *M. tuberculosis* (TBCM, encoded by Rv1885c) is assumed to play a key role in the pathogenesis of TB; however, the mechanism remains unknown.

**Methods:**

A *tbcm* deletion mutant (B∆*tbcm*) was generated by targeted gene knockout in BCG to investigate the pathogenic role of TBCM in mice or macrophages. We compared the pathogenesis of B∆*tbcm* and wild-type BCG in vivo by measuring the bacterial clearance rate and the degree of apoptosis. Promotion of the intrinsic apoptotic pathway was evaluated in infected bone marrow-derived macrophages (BMDMs) by measuring apoptotic cell death, loss of mitochondrial membrane potential and translocation of pore-forming proteins. Immunocytochemistry, western blotting and real-time PCR were also performed to assess the related protein expression levels after infection. Furthermore, these findings were validated by complementation of *tbcm* in BCG.

**Results:**

Deletion of the *tbcm* gene in BCG leads to reduced pathogenesis in a mouse model, compared to wild type BCG, by promoting apoptotic cell death and bacterial clearance. Based on these findings, we found that intrinsic apoptosis and mitochondrial impairment were promoted in B∆*tbcm*-infected BMDMs. B∆*tbcm* down-regulates the expression of Bcl-2, which leads to mitochondrial outer membrane permeabilization (MOMP), culminating in cytochrome c release from mitochondria. Consistent with this, transcriptome profiling also indicated that B∆*tbcm* infection is more closely related to altered mitochondrial-related gene expression than wild-type BCG infection, suggesting an inhibitory role of TBCM in mitochondrial dysfunction. Moreover, genetic complementation of B∆*tbcm* (C∆*tbcm*) restored its capacity to inhibit mitochondria-mediated apoptotic cell death.

**Conclusions:**

Our findings demonstrate the contribution of TBCM to bacterial survival, inhibiting intrinsic apoptotic cell death of macrophages as a virulence factor of *M. tuberculosis* complex (MTBC) strains, which could be a potential target for the development of TB therapy.

**Supplementary Information:**

The online version contains supplementary material available at 10.1186/s12929-023-00988-2.

## Background

Tuberculosis (TB) is one of the deadliest infectious diseases worldwide and causes 1.5 million deaths annually [1]. *Mycobacterium bovis* bacillus Calmette-Guérin (BCG), an attenuated strain of virulent *M. bovis*, is the only available vaccine against TB [2]. However, BCG vaccination exhibits variable efficacy in adults and can cause disseminated disease (BCG-osis), especially in immunocompromised individuals [3]. Because of the limitations of BCG, several attempts have been made to improve its efficacy and safety by identifying its virulence genes.

Programmed cell death (apoptosis), as an evolutionarily conserved defense mechanism, plays a pivotal role in the innate immune response of the host against intracellular pathogens such as *Mycobacterium tuberculosis* (Mtb) [4]. Mtb has evolved an assortment of strategies to inhibit the defense mechanisms of infected host cells, particularly macrophages that serve as the first line of innate defense against infections, which can facilitate its persistent intracellular lifestyle [5]. To date, several antiapoptotic genes of Mtb have been identified, including *nuo*G, which encodes a subunit of type-1 NADH dehydrogenase (NADH-1), tyrosine phosphatase (PtpA) and Mtb Cpn60.2 (GroEL2) [6]. Therefore, understanding the mechanisms underlying Mtb-mediated manipulation of macrophage apoptosis could provide insights for developing TB treatments and interventions.

Chorismate mutase (CM) plays a pivotal role in the shikimate pathway in bacteria or fungi and is responsible for synthesizing aromatic amino acids, including phenylalanine, tyrosine, and tryptophan, by converting chorismate into prephenate [7]. There are two types of genes encoding CMs in the Mtb genome: Rv1885c and Rv0948c. Of these CMs, secretory TBCM (encoded by Rv1885c) is considered to have an important role in TB pathogenesis, showing a low level of sequence homology with intracellular CM, Rv0948c [8, 9] Therefore, it has drawn attention as a putative drug target for TB treatment [10]. Despite the limited understanding about the pathogenic role of TBCM, we recently reported that TBCM can enhance dendritic cell (DC) maturation in bone marrow-derived DCs (BMDCs) in a TLR4-dependent manner and can be effectively used as a novel immunoadjuvant, also suggesting its potential role in Mtb pathogenesis [11].

In this study, we targeted the TBCM of the BCG Tokyo strain (BAH26192.1), which shares an identical protein sequence with the TBCM of the Mtb H37Rv strain (NP_216401.1), and elucidated the mechanism underlying the pathogenic role of TBCM in the pathogenesis of TB by deleting the TBCM gene in the BCG strain. We mainly discussed the changes in BCG-induced apoptosis according to the presence of the TBCM gene in BCG and elucidated the mechanism underlying the pathogenic role of TBCM, which could be similar to Mtb infection.

## Methods

### Bacterial growth conditions

*M. bovis* BCG strain Tokyo 172 and its mutant (B∆*tbcm*) were incubated at 37 °C in complete 7H9 broth prepared with Middlebrook 7H9 powder (Difco, 271310), 2.5% glycerol (Sigma, G9012), 0.2% Tween-80 (Sigma, P1754), and 10% ADC (Sigma, M0553) or in 7H10 agar prepared with Middlebrook 7H10 powder (Difco, 271310), 0.5% glycerol, and 10% OADC (BD, 212240). The complemented mutant (*C*Δt*bcm)* was incubated in 7H9 broth and 7H10 agar containing 100 µg/ml kanamycin (Sigma, K1876).

### Construction of *M. bovis *∆*tbcm*

The Rv1885c (TBCM) gene was deleted in wild-type *M. bovis* BCG by referring to the method in Krishnamoorthy Gopinath et al. [12]. Briefly, the plasmid for the knockout was constructed by amplifying the upstream flanking region (NC_012207.1: 2116270-2117236) using the primer pair Up_F (5ʹ-TTT AAG CTT CCG GTA GTA CAC GGT GGT GGT CAA CAC GGA C-3ʹ) and Up_R (5ʹ-TGA CGG GAT CAA CCG AAG GGT CAA TCC GGT TGT GCA TCC TTT GCC GG-3ʹ) and the downstream flanking region (NC_012207.1: 2117837-2118836) using the primer pair Down_F (5ʹ-AGG ATG CAC AAC CGG ATT GAC CCT TCG GTT GAT CCC GTC AGC C-3ʹ) and Down_R (5ʹ-AAA GGA TCC CAG GTA TGA CAG ACG TGA GCC GAA AGA TTC). Next, the amplified DNA from the upstream and downstream flanking regions was used as a template to perform the fusion of two segments with the primer pair Up_F and Down_R. The resulting product was then cloned and inserted into the p2NIL vector using HindIII (NEB, R3104L) and BamHI (NEB, R3136L) with DNA Ligation Mix (TaKaRa, 6023) according to the manufacturer’s instructions. To introduce the selection markers that were needed to select mutants, the PacI (NEB, R0547L)-digested 8-kbp fragment of pGOAL19 was ligated with the p2NIL construct in which the TBCM flanking regions were inserted to construct the final plasmid, pBTdel.

After electroporation at 2.5 kV, 25 µF, and 1000 Ω, the pBTdel plasmid, which was exposed to 100 mJ/cm^2^ of UV irradiation, was integrated into the chromosome through homologous recombination, referred to as single crossover (SCO). To select for bacteria with integrated pBTdel, we plated them on 7H10 agar supplemented with 25 µg/ml kanamycin and 50 µg/ml hygromycin (Sigma, 31282-04-9). However, due to the properties of the p2NIL vector, which lacks a mycobacterial origin of replication, bacteria that did not have pBTdel integrated into the chromosome were unable to possess antibiotic resistance when they replicated. Subsequently, we identified SCO bacteria by observing blue colonies, a result of the *lacZ* gene present in pBTdel, following treatment with 400 µL of 0.4% X-gal on an agar plate underlay. After identifying the *lac*Z-screened single crossovers, we cultured them in 7H9 broth with 25 µg/ml kanamycin and 50 µg/ml hygromycin. These cultures were then incubated on 7H10 agar with 2% sucrose (Sigma, S0389) to select for bacteria that had undergone a second crossover, referred to as a double crossover (DCO). To confirm the presence or absence of pBTdel, a counterselection method using the characteristics of *lacZ* was also employed. Colonies that did not respond to *lacZ* were confirmed to be either wild-type BCG or B∆*tbcm* through polymerase chain reaction (PCR) using the primer pair confirm_F (5ʹ- CAC ATA CGT CGA CCT GGT CAT AGA CC -3ʹ) and confirm_R (5ʹ- CCA GAC TTACAA GTG GGA AAC CCT CC-3ʹ).

### Complementation of B∆*tbcm*

To generate plasmid DNA expressing TBCM, we used the pMyong2 vector, which is a *Mycobacterium*-*E. coli* shuttle vector. The heat shock protein 65 gene (hsp65) promoter region and TBCM gene were amplified from *M. bovis* BCG. The primers used for overlap PCR were as follows: hsp65 gene (forward 5ʹ-AAA GCG GCC GCG GTG ACC ACA ACG ACG CG-3ʹ, reverse 5ʹ-GGG TAA GCA TTG CGA AGT GAT TCC TCC GGA TC-3ʹ) and TBCM gene (forward 5ʹ-TCA CTT CGC AAT GCT TAC CCG TCC ACG TGA G-3ʹ, reverse 5ʹ-AAA TCT AGA TCA GGC CGG CGG TAG-3ʹ). The amplified hsp65 gene and TBCM gene were overlapped by PCR and digested with NotI (NEB, R3189L) and XbaI (NEB, R0145L) at 37 °C overnight. The digested gene was ligated with the pMyong2 vector using a DNA ligation kit.

To generate C∆*tbcm* expressing pMyong2-*tbcm,* the plasmid was transformed into competent B∆*tbcm* cells through electroporation at 2.5 kV, 25 µF, and 1000 Ω. C∆*tbcm* was incubated on 7H10 supplemented with OADC, 0.5% glycerol and 100 µg/ml kanamycin at 37 °C for 3 weeks. PCR was conducted to confirm whether the colonies expressed the *tbcm* gene.

### Whole-genome resequencing

We commissioned whole-genome resequencing of B*∆tbcm* and its analysis to e-biogen (Seoul, South Korea). Briefly, the sequencing libraries were prepared following the manufacturer’s instructions for the TruSeq DNA Nano Library Prep Kit (Illumina). The libraries were quantified using qPCR via KAPA Library Quantification kits and assessed by the Agilent Technologies 2200 TapeStation (Agilent Technologies). The libraries were sequenced using NovaSeq™ (Illumina).

FastQC (v0.11.5) and Trimmomatic (v0.36) were applied to identify low-quality reads and reduce biases. Data were aligned using BWA (v0.7.17) with a mem algorithm referring to the genome of the NCBI RefSeq database. Duplicated reads were excluded using Sambamba (v0.6.7). Genome coverage and the ratio of mapped reads to the reference genome were determined. Variant calling was conducted using SAMtools (v.1.6) and BCFtools (v.1.6). During this step, potent single nucleotide polymorphisms and short indels with phred scores (> 30, 99.9% base call accuracy) were identified based on information from the mapped reads. Captured variants were annotated with SnpEff (v.4.3t) to estimate the effects of genetic variants.

### In vivo mouse model and infection

Seven-week-old BALB/c mice (Female) were purchased from Orient-Bio (Seoul, South Korea) and used for experiments at 8 weeks of age. Wild-type BCG and BΔ*tbcm* were diluted to 1 × 10^7^ colony forming units (CFU)/ml in PBS. In addition, 100 µL (1 × 10^6^ CFU) of wild-type BCG and B∆*tbcm* were injected intravenously into 8-week-old mice. Four weeks after injection, the mice were sacrificed.

### Cell infection

Bone marrow-derived macrophages (BMDMs) and J774A.1 cells were infected with wild-type BCG, B∆*tbcm* and C∆*tbcm* at a multiplicity of infection (MOI) of 10 in RPMI 1640 containing 10% FBS and incubated for 4 h at 37 °C and 5% CO_2_. To remove extracellular mycobacteria, infected BMDMs and J774A.1 cells were washed with PBS and incubated in fresh medium containing 10 µM amikacin for 2 h, and then the medium was changed to fresh medium with 10% FBS.

### RNA-seq data analysis

BMDMs (1 × 10^6^) were infected with wild-type BCG and B∆*tbcm* at an MOI of 10. At 2 h postinfection, total RNA from infected BMDMs was isolated using TRIzol reagent. We request RNA sequencing and analysis to e-biogen (Seoul, South Korea). QuantSeq 3ʹ mRNA-Seq reads were aligned by using Bowtie2. The alignment was generated from either the genomic assembly sequence or the representative transcript sequences. The alignment result was utilized for constructing transcripts, quantifying their abundances and identifying the differential expression of genes (DEGs). Analysis of DEGs was performed based on counts from alignments using coverage in Bedtools. To normalize the read count data, the TMM + CPM method was applied using EdgeR within R language. The classification of genes was achieved by the DAVID and Medline databases. ExDEGA (Ebiogen Inc., South Korea) was used for data mining and visualization. The raw RNA-seq data are provided in Additional file 2.

### Protein extraction and immunoblotting

Mycobacterial proteins were extracted from cultured wild-type BCG, B∆*tbcm* and C∆*tbcm* pellets. The pellets were resuspended in B-per buffer (Thermo, UD285534) containing 1 mM EDTA and protease inhibitor (Roche, 4693132001) and sonicated. The sonicated cells were centrifuged (13,000 rpm, 4 °C, 15 min), and the supernatants were collected. Recombinant TBCM (rTBCM) represents a positive control of the immunoblotting, which was applied by cloning the TBCM gene into the pET-28a vector and expressing the His-tagged (5 kDa) TBCM protein in *E. coli* Rosetta2(DE3), followed by purification using Ni–NTA affinity chromatography. In the case of total cell proteins, RIPA buffer (Thermo, 89900) containing protease inhibitor and phosphatase inhibitor (Roche, 4906845001) was used to treat infected BMDMs and J774A.1 cells (1 × 10^6^) at 18 and 24 h postinfection. These suspensions were centrifuged, and the supernatants were collected. To evaluate protein expression in mitochondria and cytosol, mitochondrial and cytosolic proteins from infected BMDMs and J774A.1 cells (2 × 10^6^) were isolated at 6 h postinfection using a mitochondrial/cytosol fractionation kit (Abcam, ab65320) following the manufacturer’s instructions. All these samples were quantified using the Bradford assay (Bio-Rad, 5000207).

The proteins were fractionated by SDS‒PAGE and transferred to nitrocellulose membranes. The membranes were blocked with 5% skim milk (232100) and incubated with primary antibodies at 4 °C overnight. The membranes were incubated with HRP-conjugated secondary antibodies (1:10,000) for 2 h at RT. The proteins were detected with ECL solution (Cytiva, RPN2232) and an Amersham Imager 600 imager. All uncropped blot images are shown in Additional file 1: Fig. S7-S9), and the antibodies used are listed in Additional file 1: Table S1.

### Flow cytometry

For the cell apoptosis analysis, infected BMDMs or J774A.1 cells (1 × 10^6^) were harvested at 24 and 48 h postinfection, and an Annexin V-FITC Apoptosis Detection Kit (Sigma, APOAF-20TST) was used to assess apoptosis. Cells were suspended in 2 µL of Annexin V-FITC conjugate and 2 µL of propidium iodide solution for 10 min at RT. The membrane potential of the mitochondria of infected BMDMs (1 × 10^6^) was measured at 24 h postinfection by using a TMRE-Mitochondrial Membrane Potential Assay Kit (Abcam, ab113852) and JC-1 (MedChemExpress, HY-15534). The FCCP positive control was prepared by adding 20 µM FCCP for 10 min prior to TMRE staining and incubating the cells with 400 nM TMRE for 15 min. mtROS measurement was performed by using MitoSox™ Red microbial superoxide indicator (Invitrogen, M36008). Infected BMDMs (1 × 10^6^) were harvested at 3 h postinfection and incubated with 3 µM MitoSox™ Red reagent for 30 min at 37 °C. Each assay was conducted according to the manufacturer’s protocol and analyzed by a BD LSRFortessa™ X-20. The obtained data were analyzed using FlowJo 10 software.

### Immunocytochemistry (ICC) and confocal imaging

BMDMs or J774A.1 cells (1 × 10^5^) were seeded in 2-well chamber slides (Thermo Scientific, 154461). A TUNEL assay kit (Abcam, ab66108) was used to detect DNA fragmentation and apoptotic cells. Infected BMDMs were fixed with 4% PFA for 20 min and incubated with 70% ethanol for 30 min. The staining solution was added following the manufacturer’s protocol, and the sample was incubated at 37 °C for 60 min. For detection of the mitochondrial membrane potential, JC-1 (Medchem Express, HY-15534) staining was performed with 1 ml of working solution, and the sample was incubated at 37 °C for 20 min. Cytochrome c release and translocation of BAX were detected with mitochondrial labeling. Infected BMDMs were harvested at 8 h postinfection, stained with 500 nM Mitotracker**®** Deep Red FM – special packaging (Invitrogen, 22426) for 45 min and fixed with 4% PFA. Before cytochrome C and BAX staining, cells were permeabilized with 0.1% Triton X-100 (in PBS) for 20 min at RT and blocked with 1% BSA (in PBS) solution. A 1:200 dilution of Alexa Fluor**®** 488 anti-Cytochrome C antibody (BioLegend, 612308) and Alexa Fluor**®** 488 anti-BAX antibody (BioLegend, 633604) was applied, and the sample was incubated at 4 °C overnight. After washing with PBS, stained cells were mounted with Vectashield**®** antifade mounting medium with DAPI (Vetor laboratories, H-1200–10). Images were obtained by confocal microscopy (Olympus, Fluoview FV3000).

### Seahorse XF24 cell mito stress test

To evaluate cell mitochondrial stress, a Seahorse XF2 analyzer (Agilent, 102342-100) and XF Cell Mito Stress Test Kit (Agilent, 103015-100) were used. The drugs used in this assay were as follows: oligomycin (2.5 µM), FCCP (2 µM) and rotenone/antimycin A (0.5 µM).

At 2 h postinfection, the media of infected BMDMs (5 × 10^5^) was replaced with XF RPMI base medium (Agilent, 103576-100) containing 300 mg/L L-glutamine (Thermo, 25030081) and 2000 mg/L D-glucose (Welgene, LS001-01) and incubated in the absence of CO_2_ for 1 h. After 1 h of incubation, a cell mito stress test was conducted.

### Statistical analysis

All experimental data were analyzed with GraphPad Prism 10. Significant differences among multiple experimental groups were identified by one- or two-way ANOVA with Tukey’s multiple comparison test, and Student’s t test was applied to compare pairs of experimental groups. The data are presented as the mean ± SEM, and statistical significance is indicated with asterisks as follows: *P < 0.05, **P < 0.01, and ***P < 0.001.

* Detailed methods are provided in Additional file 1: Additional materials and Table S2.

## Results

### Deletion of *tbcm* in *Mycobacterium bovis* BCG

**Fig. 1:**
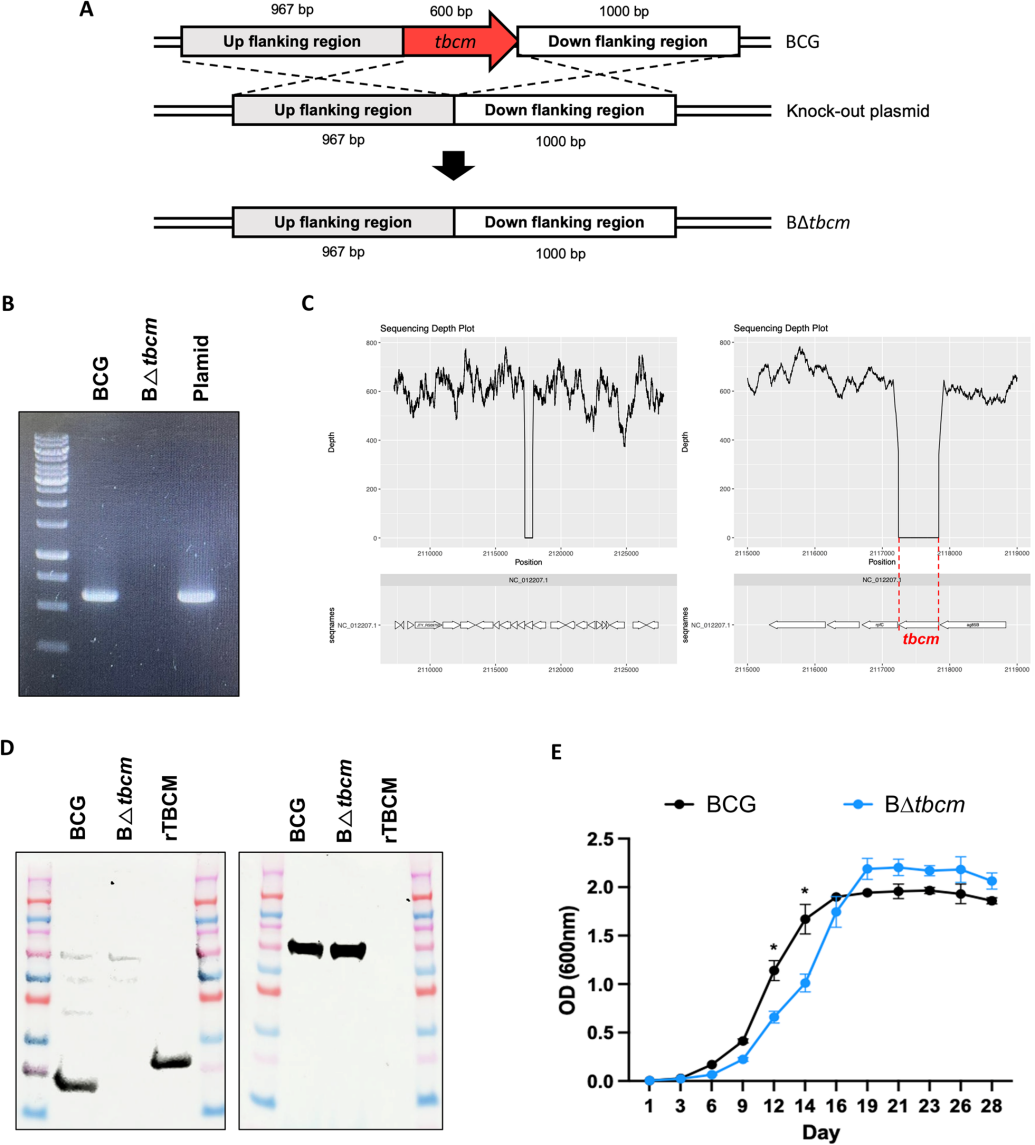
Generation of the *Mycobacterium bovis* BCG *tbcm* deletion mutant. **A** Schematic diagram of the *tbcm* gene deletion strategy. **B** Whole-gel image of polymerase chain reaction (PCR) confirmation of *tbcm* gene (600 bp) expression in wild-type BCG (BCG) and BΔ*tbcm*. **C** Whole-genome sequencing result of gDNA isolated from BΔ*tbcm*. The *tbcm* gene deletion was confirmed by a depth level of 0 in the sequencing depth plot. **D** Uncropped blot image of TBCM (left) and hsp65 (right) protein expression in BCG and BΔ*tbcm*. rTBCM represents recombinant TBCM. **E** Graphs comparing the growth curves of BCG (gray) and BΔ*tbcm* (blue). The growth curve of each mycobacterial strain was drawn by measuring the OD_600_ at each time point. All data were collected in at least three independent experiments and are presented as the mean ± SEM. The results were analyzed for statistical significance by two-way ANOVA with Tukey’s multiple comparison test. (*P < 0.05, **P < 0.01, ***P < 0.001). *BCG* wild-type BCG, BΔ*tbcm*, BCG Δ*tbcm* mutant

TBCM (Rv1885c) has been considered a potential target for vaccines that might exert pathogenic effects on TB [10]. To investigate the function of TBCM in pathogenesis, we first deleted the *tbcm* gene in *M. bovis* BCG (NC_012207.1: 2,117,239–2,117,838), which is completely identical to the TBCM of *M. tuberculosis* (Rv1885c), through mycobacterial electroporation and homologous recombination (Fig. 1A). The removal of the 600 bp *tbcm* gene in B∆*tbcm* was confirmed by PCR (Fig. 1B). The genomic DNA of B∆*tbcm* was resequenced against *M. bovis* BCG str. Tokyo 172, and the mapping depth surrounding the deleted region is represented in the graph (Fig. 1C). The graphs show that the *tbcm* region was not read, which confirms that *tbcm* was deleted in the gDNA of BCG. Additionally, we verified abrogation of TBCM production in B∆*tbcm*, while the mRNA and the protein were expressed in wild-type BCG (Additional file 1: Fig. S1 and Fig. 1D). To determine whether deletion of TBCM affects mycobacterial growth, we compared the growth rates of wild-type BCG and B∆*tbcm*. Although there was no significant difference during the stationary phase, wild-type BCG grew faster than B∆*tbcm* in the log phase, indicating that deletion of *tbcm* affects growth (Fig. 1E).

### TBCM is a virulence factor of BCG that inhibits apoptotic cell death in vivo

**Fig. 2:**
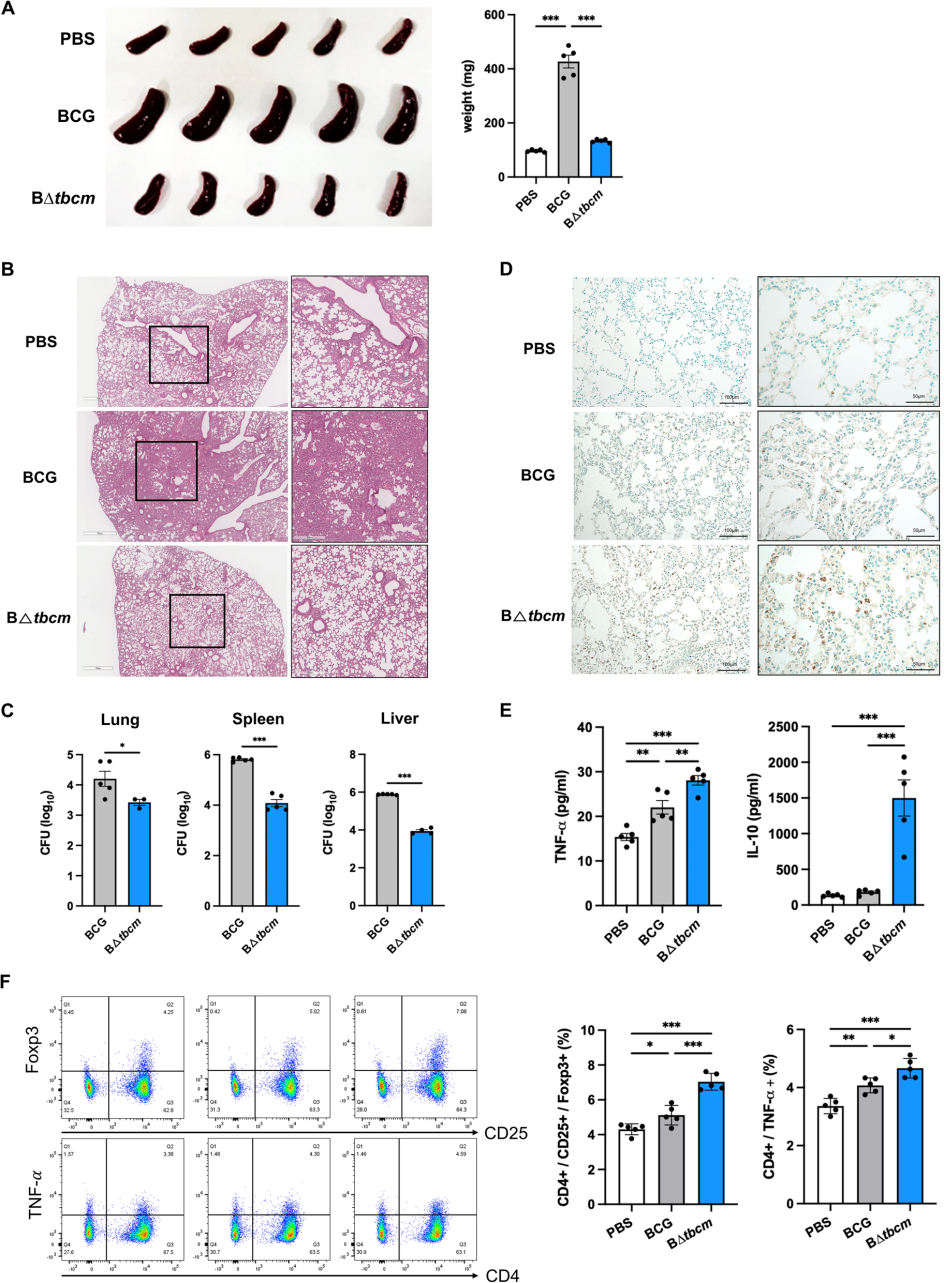
Deletion of *tbcm* enhances apoptosis in a mouse model. Female BALB/c mice (n = 5 per group) were infected with BCG or BΔ*tbcm* (1 × 10^6^ CFU) intravenously and sacrificed at 4 weeks postinfection. **A** Comparison of spleen size (left) and weight (right). **B** Histopathological images (H&E staining) of lung tissues (scale bars, 300 $\upmu$m or 700 $\upmu$m) **C** The bacterial burdens in lungs, spleens and livers were measured by the CFU assay. **D** Apoptotic cells in lung tissues were detected by the TUNEL assay (scale bars, 50 $\upmu$m or 100 $\upmu$m). **E** Splenocytes were isolated from infected mice and incubated for 3 days. The levels of TNF-$\mathrm{\alpha }$ and IL-10 were detected in the culture supernatants of splenocytes by ELISA.) CD25 + /Foxp3 + regulatory T cell and CD4 + /TNF-$\mathrm{\alpha }$+ T-cell subsets were analyzed in splenocytes by flow cytometry. All data were collected in at least three independent experiments and are presented as the mean ± SEM. The results were analyzed for statistical significance by two-tailed Student’s t test (**C**) or one-way ANOVA with Tukey’s multiple comparison test (**A**, **E**, **F**). (*P < 0.05, **P < 0.01, ***P < 0.001). BCG, wild-type BCG; BΔ*tbcm*, BCG Δ*tbcm* mutant

To assess the role of TBCM in pathogenesis in an in vivo system, we injected wild-type BCG and B∆*tbcm* intravenously to infect BALB/c mice. At 4 weeks postinfection, the weight of the spleen of mice infected with wild-type BCG increased significantly, whereas the mice infected with B∆*tbcm* showed alleviation of splenomegaly (Fig. 2A). The histopathology of lung tissues showed alleviated inflammatory lesions in mice infected with B∆*tbcm* compared to mice infected with wild-type BCG (Fig. 2B). Additionally, deletion of *tbcm* resulted in a lower bacterial burden in the spleen, lungs and liver than that observed with wild-type BCG, reflecting the resolution of infection (Fig. 2C). It is known that inhibition of apoptosis is a virulence strategy of pathogenic mycobacteria that reduces the killing of the bacteria in the host [13]. To determine the correlation of the virulence and apoptosis inhibition capacity of each strain, we performed TUNEL staining on the lung tissues to assess DNA fragmentation as a marker of apoptotic cell death [14]. We found increased TUNEL-positive cells in B∆*tbcm*-infected lungs compared with wild-type BCG-infected lungs, indicating higher levels of apoptosis in the absence of *tbcm* in BCG (Fig. 2D). On the other hand, we found enhanced TNF-$\alpha$ and IL-10 levels in response to B∆*tbcm* infection (Fig. 2E). Consistent with this finding, TNF-$\alpha$-expressing CD4 T cells and regulatory T cells were also identified at a high frequency in the spleen of the B∆*tbcm* group (Fig. 2F). In particular, heightened IL-10 levels are the result of counteraction to inflammatory cytokines that contribute to alleviating pulmonary inflammation [15]. Collectively, the findings showed that deletion of *tbcm* in BCG abrogates its ability to inhibit apoptosis, implying that TBCM plays an important role in the survival of BCG in the host.

### TBCM, an antiapoptotic virulence factor of BCG, regulates caspase expression in macrophages

**Fig. 3:**
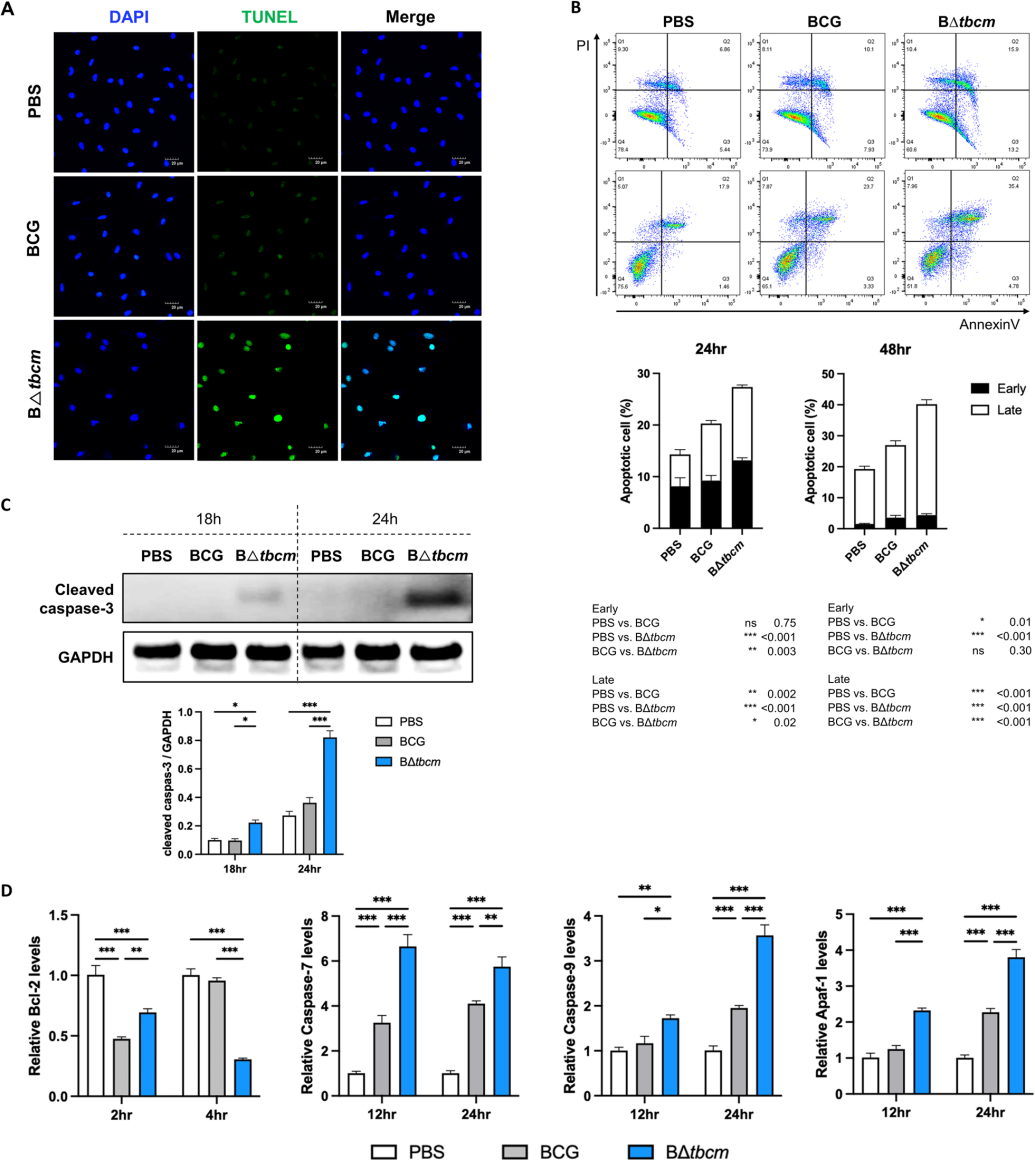
TBCM regulates apoptotic cell death in macrophages. BMDMs were isolated from female BALB/c mice and infected with BCG or BΔ*tbcm* at an MOI of 10. **A** Apoptotic cells were detected by the TUNEL assay at 24 h postinfection. **B** Early or late apoptotic cell death was quantified by the Annexin V/PI assay at 24 and 48 h postinfection, and the statistical significance is shown in the table. **C** The expression level of cleaved caspase-3 was analyzed by western blotting at 18 and 24 h postinfection. **D** The mRNA expression levels of apoptosis-associated genes were measured by real-time PCR. The Bcl-2, caspase-7, caspase-9 and Apaf-1 mRNA expression levels were measured in infected BMDMs at 2 and 4 h or 12 and 24 h postinfection. All data were collected in at least three independent experiments and are presented as the mean ± SEM. The results were analyzed for statistical significance by two-way ANOVA with Tukey’s multiple comparison test. (*P < 0.05, **P < 0.01, ***P < 0.001). BCG, wild-type BCG; BΔ*tbcm*, BCG Δ*tbcm* mutant

Macrophages act as a frontline of host defense against Mtb infection, especially in the lung alveoli, promoting bacterial clearance by apoptosis [16]. To further verify the dependence on TBCM in BCG-induced apoptosis, we conducted a TUNEL assay in BMDMs infected with each strain. We found a significant increase in TUNEL-positive cells in B∆*tbcm*-infected BMDMs compared to wild-type BCG-infected BMDMs (Fig. 3A). In addition, the levels of cell death were quantified by Annexin V/PI double staining at 24 and 48 h [17]. At 24 h postinfection, Annexin V + /PI- staining indicated early apoptotic cells, accounting for 13.15 $\pm$ 1.45% of the cells in the B∆*tbcm* group and 9.23 $\pm$ 2.7% in the wild-type BCG group. Late apoptotic cells were defined by the Annexin V + /PI + population, representing 14.21 $\pm$ 0.9% of the cells in the B∆*tbcm* group and 11.05 $\pm$ 1.5% in the wild-type BCG group. Furthermore, 48 h after infection, 35.61 $\pm$ 2.41% of the cells in the B∆*tbcm* group and 23.88 $\pm$ 2.76% of the cells in the wild-type BCG group underwent late apoptosis (Fig. 3B). Consistent with our in vivo results, we confirmed enhanced TNF-$\alpha$ secretion levels in response to B∆*tbcm* infection and an increase in IL-10 production as a counterbalance (Additional file 1: Fig. S2). These results demonstrated that the proportion of apoptotic cells (total Annexin V +) in B∆*tbcm*-infected BMDMs was notably higher than that in BMDMs infected with wild-type BCG, indicating that TBCM contributes to the apoptotic resistance of BCG in macrophages.

Macrophages undergo apoptosis in response to Mtb infection to limit mycobacterial growth in a caspase-dependent manner [18]. To confirm that deletion of *tbcm* is responsible for caspase-dependent apoptotic cell death, we assessed the activation level of caspase-3, which serves as an executioner for the final apoptotic signal in both the intrinsic and extrinsic pathways of apoptosis [19]. We identified an increased level of cleaved caspase-3 in B∆*tbcm*-infected BMDMs at 18 h and 24 h postinfection, indicating the promotion of the apoptotic process by deletion of *tbcm* (Fig. 3C). We also found a reduced level of Bcl-2, known for regulating caspase-3 activation and apoptosis-associated processes, in B∆*tbcm*-infected BMDMs compared with BMDMs infected with wild-type BCG [20]. Furthermore, an increase in caspase-7 and caspase-9 expression levels was identified in response to B∆*tbcm* infection. Consistent with these observations, the enhanced expression level of Apaf-1, which is a known upstream molecule for the activation of caspase-9 [21], was observed in B∆*tbcm*-infected BMDMs (Fig. 3D). These results demonstrate that deletion of *tbcm* increases susceptibility to apoptosis in macrophages, indicating that TBCM regulates BCG-induced apoptosis by suppressing the expression of caspase-associated molecules in macrophages.

### B∆*tbcm* enhances intrinsic apoptosis by inducing mitochondrial dysfunction

**Fig. 4:**
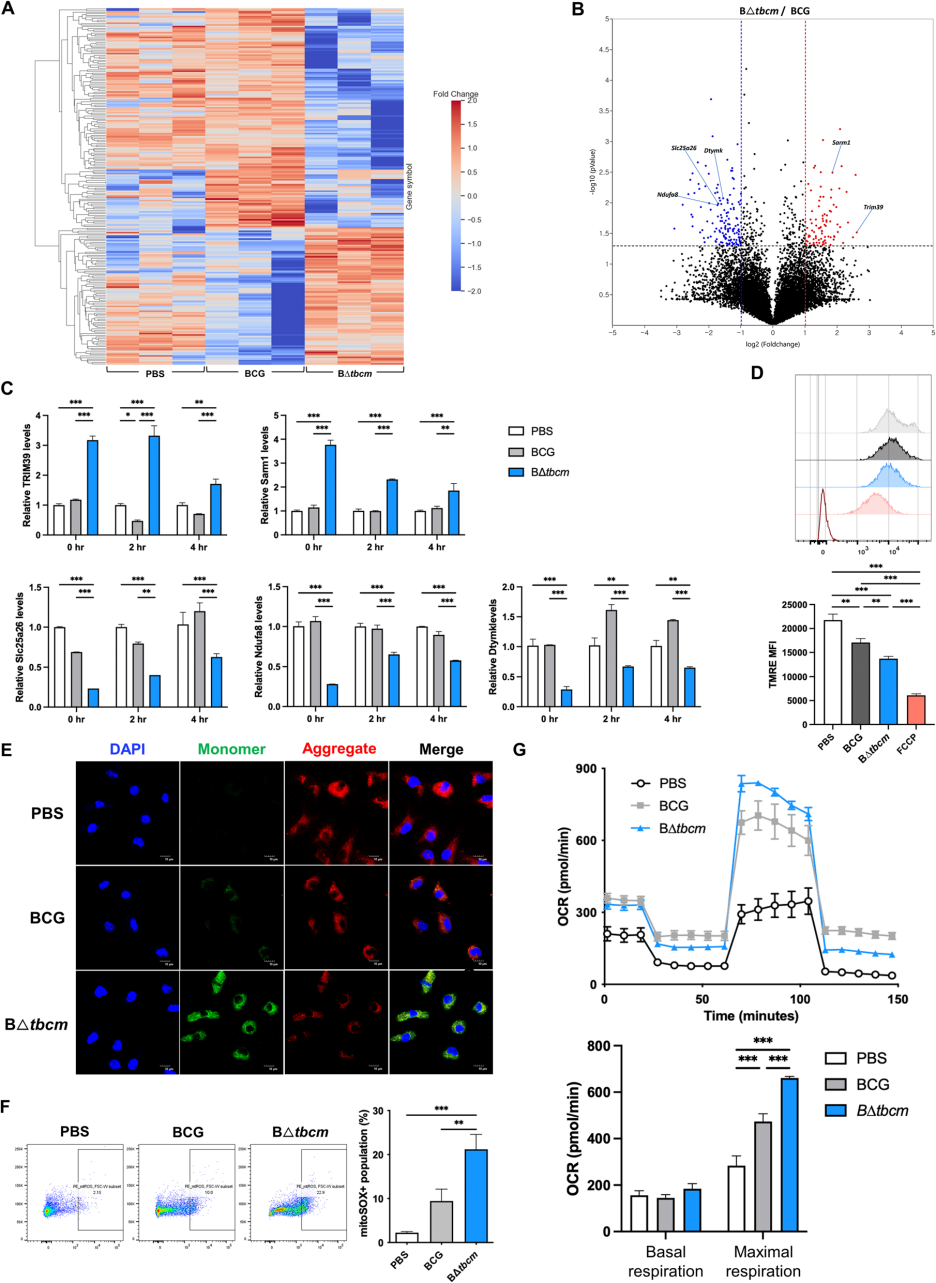
BΔ*tbcm* induces mitochondrial dysfunction in macrophages. BMDMs (BALB/c, female) were infected with BCG or BΔ*tbcm* at an MOI of 10. **A**, **B** DEGs between BCG- and BΔ*tbcm*-infected BMDMs at 2 h postinfection. **A** Heatmap presenting the DEGs in BCG- or B∆*tbcm*-infected BMDMs and (**B**) a volcano plot (blue and red dots show DEGs with decreased and increased expression, respectively). **C** The upregulated (*TRIM39*, *SARM1*) and downregulated (*SLC25A26, NDUFA8, DTYMK*) gene expression levels were measured by real-time PCR at 0, 2 and 4 h postinfection. **D** The mitochondrial membrane potential (ΔΨ_m_) was measured by TMRE staining and analyzed by flow cytometry at 24 h postinfection. **E** Fluorescence microscopy images representing ΔΨ_m_ at 24 h post infection by JC-1 staining. **F** mtROS levels were measured by mitoSOX staining at 2 h postinfection. **G** Respiratory profiles (oxygen consumption rate) of infected BMDMs were analyzed by an Agilent Seahorse XFe24 analyzer 2 h postinfection (left). Bar graphs showing quantification of basal respiration and maximal respiration derived from the respiratory profile (right). All data were collected in at least three independent experiments and are presented as the mean ± SEM. The results were analyzed for statistical significance by two-way (**C**) or one-way (**D**, **F**, **G**) ANOVA with Tukey’s multiple comparison test. (*P < 0.05, **P < 0.01, ***P < 0.001). *BCG* wild-type BCG, BΔ*tbcm*, BCG Δt*bcm* mutant

Both intrinsic and extrinsic apoptotic pathways are responsible for Mtb-induced apoptosis in macrophages [22]. In addition, activation of caspase-9 and Apaf-1 can be classified as a signature of the intrinsic apoptotic pathway, as we observed in B∆*tbcm*-infected BMDMs [23]. Therefore, we hypothesized that deletion of *tbcm* in BCG is associated with mitochondrial dysfunction, thereby resulting in intrinsic apoptosis. We interrogated the mitochondrial transcriptomic profiles of B∆*tbcm-* or wild-type BCG-infected BMDMs. Transcriptome analysis revealed that genes were differentially expressed between B∆*tbcm-*infected BMDMs and wild-type BCG-infected BMDMs (Fig. 4A). We found 223 differentially expressed genes (DEGs) between infected BMDMs (log_2_fold change $\ge$ 2.0 and p value < 0.05), including 86 upregulated and 137 downregulated genes (Fig. 4B). *TRIM39* (+ 6.095-fold), which was more highly expressed in BMDMs infected with B∆*tbcm* than in BMDMs infected with wild-type BCG, is known to promote cytochrome c release, which can be induced by impairments in mitochondrial function [24, 25]. Likewise, the expression level of *SARM1* (+ 3.602-fold) was also elevated, which was activated by mitochondrial dysfunction [26]. Furthermore, compared to the wild-type BCG group, the transcriptome of the B∆*tbcm* group showed downregulated genes, including *SLC25A26* (− 3.276-fold), *NDUFA8* (− 3.324-fold) and *DTYMK* (− 2.931-fold); the absence of these genes is associated with mitochondrial disease (Fig. 4B) [27–29]. Specifically, we confirmed that deletion of *tbcm* significantly increased the expression of genes related to mitochondrial dysfunction, such as *TRIM39* and *SARM1*, until 4 h postinfection, which is a relatively early stage of infection, and suppressed the expression of *SLC25A26, NDUFA8* and *DTYMK*, which are associated with maintaining mitochondrial homeostasis (Fig. 4C).

Mitochondrial dysfunction is known to mediate the loss of mitochondrial membrane potential (ΔΨ_m_) and excessive mitochondrial reactive oxygen species (mtROS) production, which can lead to apoptosis [30]. To identify impaired mitochondria in infected BMDMs, we analyzed ΔΨ_m_ via flow cytometry using TMRE staining, which permeates healthy mitochondria with high ΔΨ_m_ [31]. B∆*tbcm*-infected BMDMs exhibited lower TMRE fluorescence than wild-type BCG-infected BMDMs, indicating dissipation of ΔΨ_m_ (Fig. 4D). We also used JC-1 dye to quantify changes in the ΔΨ_m_ of mitochondria in response to B∆*tbcm* infection [32]. As expected, B∆*tbcm*-infected BMDMs showed increased nonaggregated JC-1 monomers compared to wild-type BCG-infected BMDMs after 24 h of infection (Fig. 4E). During mitochondrial dysfunction, oxidative stress triggers the induction of mtROS, byproducts of mitochondrial metabolism, to regulate cellular homeostasis, which leads to proinflammatory cytokine production [33, 34]. Indeed, we observed excessive mtROS production in response to B∆*tbcm* infection at 3 h postinfection (Fig. 4F). Furthermore, to validate that altered mitochondrial respiration was associated with mtROS accumulation under B∆*tbcm* infection, we assessed the oxygen consumption rate (OCR) by the Seahorse assay [35]. Although the basal respiration was not significantly different between B∆*tbcm*- and wild-type BCG-infected BMDMs, the maximal respiration was increased under B∆*tbcm* infection, indicating the progression of impaired mitochondrial respiration (Fig. 4G). These findings suggest that TBCM participates in the suppression of BCG-induced intrinsic apoptosis by sustaining related gene expression and mitochondrial homeostasis in macrophages.

### Infection with B∆*tbcm* aggravates MOMP in macrophages, resulting in mitochondrial dysfunction

**Fig. 5:**
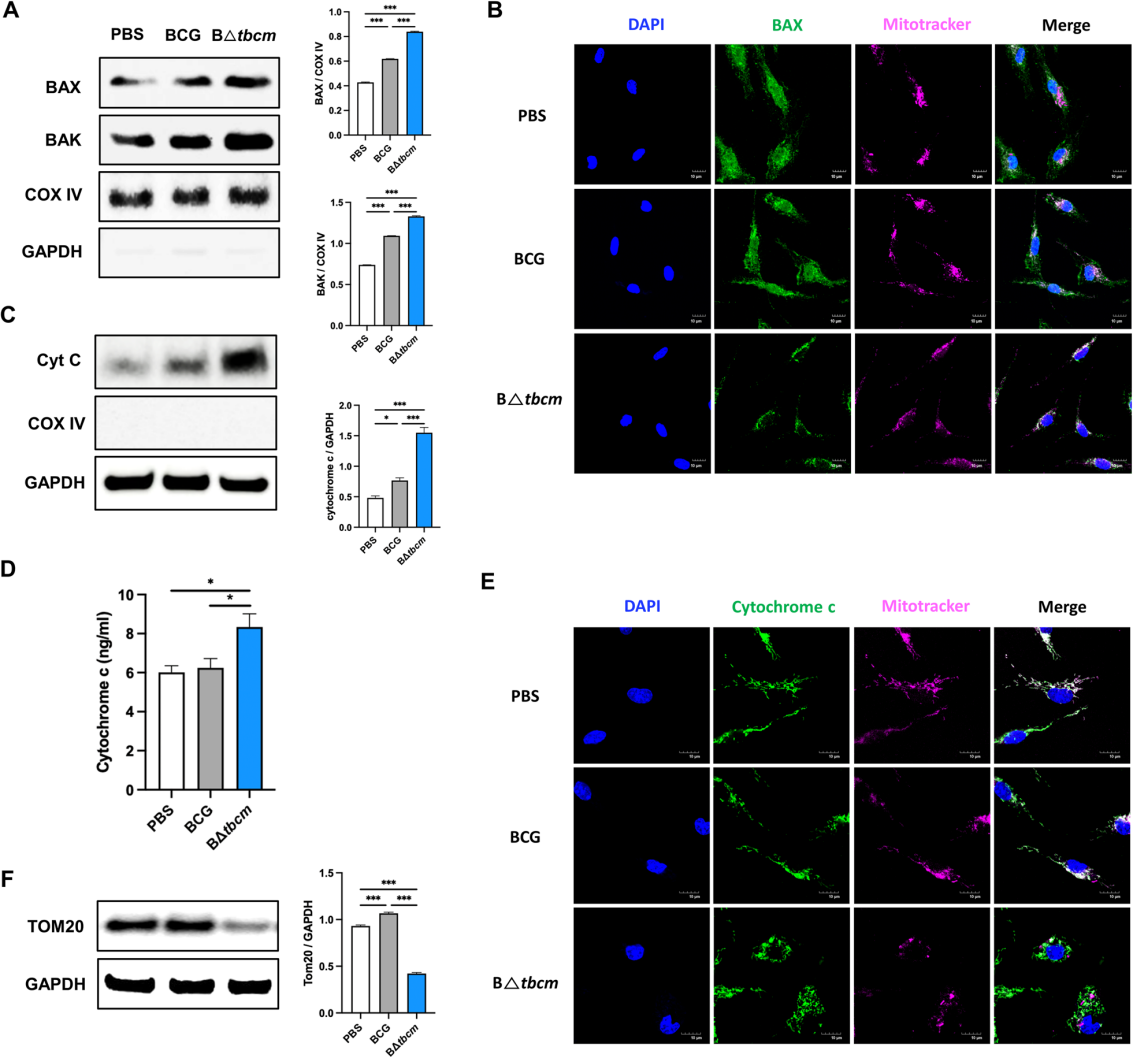
Deletion of *tbcm* exacerbates MOMP and cytochrome c release from mitochondria. BMDMs (BALB/c, female) were infected with BCG or BΔ*tbcm* at an MOI of 10. **A** Expression levels of BAX and BAK were measured in the mitochondrial fraction of infected BMDMs at 6 h postinfection by western blotting. **B** Infected BMDMs were labeled with BAX and MitoTracker mitochondria 6 h postinfection. Confocal imaging of BAX translocation from the cytosol. **C** Cytosolic cytochrome c was detected by western blotting. **D** The release of cytochrome c was measured by ELISA in the cytosolic fraction at 8 h postinfection. **E** Infected BMDMs were stained with cytochrome c and MitoTracker at 8 h postinfection. Cytosolic cytochrome c was detected by confocal microscopy. **E** Western blotting of Tom20 expression levels in total cell lysates at 18 h postinfection. All data were collected in at least three independent experiments and are presented as the mean ± SEM. The results were analyzed for statistical significance by one-way ANOVA with Tukey’s multiple comparison test (*P < 0.05, **P < 0.01, ***P < 0.001). BCG, wild-type BCG; BΔ*tbcm*, BCG Δt*bcm* mutant

The intrinsic apoptotic pathway is determined by mitochondrial outer membrane permeabilization (MOMP), which centers on the release of cytochrome c by the translocation of pore-forming proteins to mitochondria, facilitating mitochondrial dysfunction [36]. We identified enhanced mitochondrial Bax and Bak levels at 6 h postinfection with B∆*tbcm* compared to wild-type BCG (Fig. 5A). Corroborating this finding, we also observed that highly accumulated Bax protein localized to the mitochondria in B∆*tbcm*-infected BMDMs (Fig. 5B). We next measured the increase in cytochrome c release from the mitochondria of B∆tbcm-infected BMDMs. Indeed, we identified the induction of cytosolic cytochrome c at 8 h postinfection in response to B∆*tbcm* infection compared to wild-type BCG infection (Fig. 5C, D). Additionally, double staining of cytochrome c and mitochondria (MitoTracker) in B∆*tbcm*-infected BMDMs detected cytochrome c dispersal from mitochondria (Fig. 5E). Following MOMP induction, damaged mitochondria were identified in B∆*tbcm*-infected BMDMs via the reduced fluorescence intensity of MitoTracker Deep Red and the expression level of TOM20, which is known as an outer membrane protein of mitochondria (Fig. 5E, F) [37]. These results indicate that deletion of *tbcm* promotes MOMP in macrophages undergoing BCG-induced apoptosis.

### Genetic complementation of B∆*tbcm* restores its ability to inhibit apoptosis

**Fig. 6:**
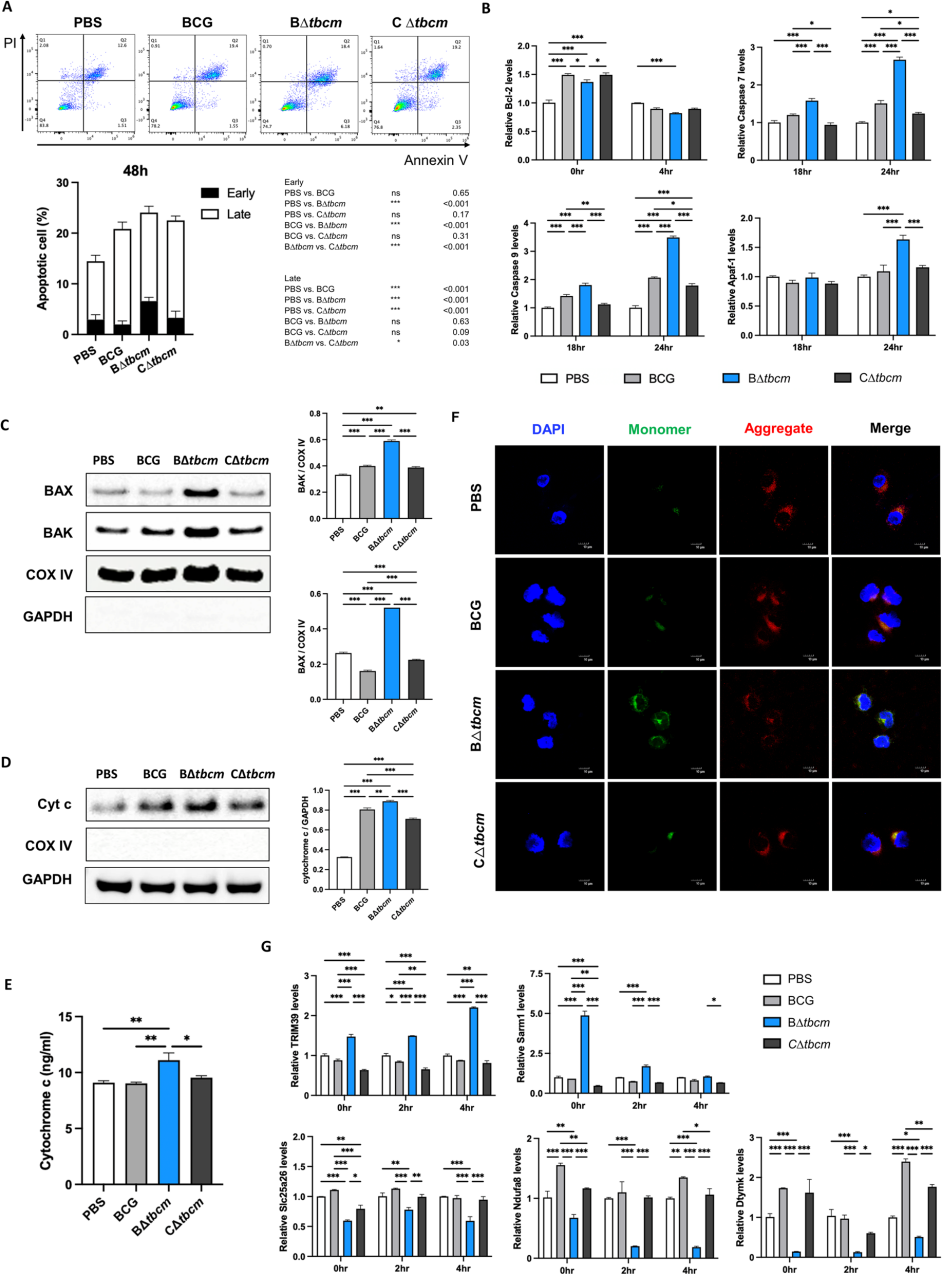
Complementation of the *tbcm* gene restored the inhibition of intrinsic apoptosis. J774A.1 cells were infected with BCG, BΔ*tbcm* or CΔ*tbcm* at an MOI of 10. **A** Apoptotic cells were quantified by the Annexin V/PI assay at 48 h postinfection, and the statistical significance is shown in the table. **B** Bcl-2, caspase-7, caspase-9 and Apaf-1 mRNA expression levels were measured by real-time PCR at 0, 4 h or 12 and 24 h postinfection. **C** Expression levels of BAX and BAK were detected in the mitochondrial fraction by western blotting. **D** Cytochrome c in the cytosolic fraction was detected at 6 h postinfection by western blotting. **E** Cytosolic cytochrome c was measured by ELISA at 8 h postinfection. **F** Cells were stained with JC-1 dye at 24 h postinfection. **G** Restored *TRIM39, SARM1, SLC25A26, NUFA8* and *DTYMK* gene expression levels were confirmed after *tbcm* complementation at 0, 2 and 4 h postinfection. All data were collected in at least three independent experiments and are presented as the mean ± SEM. The results were analyzed for statistical significance by two-way (**A**, **B**, **G**) or one-way (**C**, **D**, **E**) ANOVA with Tukey’s multiple comparison test. (*P < 0.05, **P < 0.01, ***P < 0.001). BCG, wild-type BCG; BΔ*tbcm*, BCG Δt*bcm* mutant; CΔ*tbcm,* complemented mutant

To validate the antiapoptotic role of TBCM, we recovered the mutant by complementation with pMyong2, a *Mycobacterium-E. coli* shuttle vector to enhance delivered gene expression in B∆*tbcm* (Additional file 1: Fig. S3) [38]. First, the restored capacity of the complemented mutant (C∆*tbcm*) to inhibit apoptosis was analyzed by infecting C57BL/6 mice. Mice infected with wild-type BCG or C∆*tbcm* showed increased spleen size and weight, while those infected with B∆*tbcm* showed reduced splenomegaly (Additional file 1: Fig. S4A). In line with this finding, the bacterial burden was significantly reduced in the B∆*tbcm* group compared with the wild-type BCG group, with C∆*tbcm* still showing increased bacterial burden (Additional file 1: Fig. S4B). Consistently, C∆*tbcm-*infected mice showed more aggravated lung inflammation and decreased apoptotic cell death in the lungs similar to wild-type BCG, contrasting with those of B∆*tbcm*, indicating restored apoptotic inhibition by TBCM (Additional file 1: Fig. S4C, D). Additionally, in response to C∆*tbcm* infection, we found a decrease in both IL-10 and TNF-$\alpha$ levels with the frequency of lower regulatory T cells and TNF-$\alpha$-expressing CD4 T cells compared to B∆*tbcm* (Additional file 1: Fig. S4E, F). These results corroborated the findings in Fig. 2, and both of these sets of experiments suggest an important role of TBCM in inhibiting apoptotic cell death in a mouse model. Next, we quantified the degree of apoptosis in J774A.1 cells. At 48 h postinfection, we identified an increase in early apoptotic cells in B∆*tbcm*-infected macrophages but not in those infected with C∆*tbcm* (Fig. 6A). In addition, the percentage of macrophages in early apoptosis after infection with wild-type BCG or C∆*tbcm* was not significantly different. Increased apoptosis resulted in lower CFU in B*∆tbcm* compared to wild-type BCG or C*∆tbcm-*infected BMDMs (Additional file 1: Fig. S5). To verify the inhibition of caspase-dependent intrinsic apoptosis by complementation of *tbcm*, we analyzed the related gene expression levels and the degree of mitochondrial dysfunction in macrophages. C∆*tbcm* did not alter the expression levels of Bcl-2, caspase-7/9 and Apaf-1 compared to wild-type BCG (Fig. 6B). Moreover, after *tbcm* complementation, we found restored pore-forming protein levels in the mitochondrial fraction that were approximately the same as those in the wild-type BCG group (Fig. 6C), which resulted in a reduction in cytochrome c release from mitochondria, implying the existence of an apparent virulence mechanism that operates in the unattenuated strain (Fig. 6D, E).

Consistent with these observations, the C∆*tbcm* strain showed moderately reduced ΔΨ_m_ with a slight induction of JC-1 monomers, which presented lower fluorescence than B∆*tbcm* (Fig. 6F), and the TOM20 levels also showed a slight recovery in C∆*tbcm*-infected macrophages (Additional file 1: Fig. S6). To investigate whether the complementation of *tbcm* led to recovery of altered mitochondrial gene expression, we evaluated mRNA expression in infected macrophages. Full complementation was observed for the expression levels of *TRIM39, SARM1, SLC25A26, NDUFA8* and *DTYMK* by infection with C∆*tbcm*, which showed similar trends as wild-type BCG infection (Fig. 6G). In conclusion, these observations strengthen our hypothesis that TBCM plays a pivotal role in resistance to BCG-induced apoptosis by maintaining homeostasis of mitochondria in the host cell.

## Discussion

*M. tuberculosis* complex (MTBC) members, including *M. tuberculosis* and *M. bovis* BCG, have two types of CM genes in their genomes, namely, an intracellular CM that is common in other bacteria and a secreted CM (TBCM) that is unique to strains within the genus *Mycobacterium* [39], suggesting a distinct role of the latter in their pathogenesis and evolution. Here, we report that deletion of *tbcm* in BCG, a member of the MTBC, disrupts mitochondrial homeostasis in infected murine macrophages (Fig. 4), which subsequently leads to apoptotic cell death (Figs. 2, 3), resulting in virulence attenuation. This highlights a contributory role of TBCM in the pathogenesis of MTBC members as a new virulence factor inhibiting apoptosis. There are noteworthy findings regarding the role of TBCM in MTBC pathogenesis described in this study.

Our RNA-seq analysis indicated that deletion of *tbcm* advances the disruption of mitochondrial homeostasis in murine macrophages (Fig. 4A, B). Subsequently, we also found that B∆*tbcm* can enhance mitochondrial dysfunction via the loss of mitochondrial membrane potential (ΔΨ_m_) (Fig. 4D, E) and excessive respiration, which leads to mitochondrial reactive oxygen species (mtROS) accumulation (Fig. 4F, G), consequently leading to apoptotic cell death in infected macrophages. These results suggest that TBCM could contribute to maintaining mitochondrial homeostasis in infected macrophages under host-mediated insults during infection, such as mtROS, and subsequently create a protective niche for survival by inhibiting apoptotic cell death.

It was recently reported that mitochondrial dysfunction in macrophages is linked to dampened inflammation via increased production of inflammatory cytokines such as TNF-α [40], which in turn leads to further strengthening of apoptotic cell death in infected macrophages via TNF receptor-mediated signaling [41]. Paradoxically, this enhancement of inflammatory cytokine production could also lead to increased production of IL-10 as a mechanism of negative feedback [42], which could consequently counteract BCG-mediated inflammation under infection in mice or humans in vivo. Indeed, we found that compared to wild-type BCG infection, infection of mice with B∆*tbcm* led to reduced inflammation in lung tissue, possibly via an increased regulatory T-cell subset followed by enhanced IL-10 production (Fig. 2E, F).

The limitation of our study is that although we confirmed the role of TBCM in promoting intrinsic apoptosis in macrophages, we did not determine the extrinsic pathway in BCG-induced apoptotic cell death. Since it is known that Mtb maneuvers both mitochondrial intrinsic apoptosis and TNF-$\alpha$-triggered extrinsic apoptotic pathways [43], it is necessary to investigate whether TBCM is also involved in BCG-induced extrinsic apoptosis. Notably, we found an increased level of TNF-$\alpha$ by infection with B∆*tbcm* in both in vivo and ex vivo assays, which might suggest a potent role of TBCM in inhibiting extrinsic apoptosis as well. Additionally, in this study, we did not demonstrate the secreted chorismite mutase activity on inhibiting macrophage apoptosis. We hypothesized the enzymatic activity of TBCM in terms of macrophage apoptosis based on its structure. The secretory form of TBCM (Rv1885c) is a biological homodimer, and TBCM contains a proline-rich fragment that connects two polypeptide segments to form a dimer. Therefore, the proline-rich surface of TBCM may be involved in binding to macrophage surface receptors [44]. Based on this perspective, we assume that the secretory form of TBCM might bind to receptors that can inhibit apoptotic cell death in macrophages. However, this issue remains to be further examined in future studies.

BCG vaccination has limited efficacy in adolescent and adult populations, so a new strategy to potentiate the efficacy of this vaccine is needed [45]. Since apoptosis has been reported to boost TB vaccine efficacy by enhancing innate or adaptive immune responses against TB antigens, BCG variants with gene deletion or recombinant BCG, which can potentiate the capacity of the wild-type BCG strain to enhance apoptotic cell death, have been developed as novel candidate TB vaccines [46]. Therefore, B∆*tbcm*, which is newly introduced in this study, can be used to enhance apoptotic cell death in infected antigen-presenting cells and applied as an alternative live TB vaccine.

## Conclusions

This study identified the pathogenic role of TBCM by deletion mutants. B∆*tbcm* induces translocation of pore-forming proteins to mitochondria, leading to intrinsic apoptotic cell death by regulating Bcl-2 expression in macrophages. These findings indicate that TBCM contributes to bacterial survival in host cells by inhibiting apoptotic cell death and sustaining mitochondrial homeostasis as a virulence factor of MTBC strains. Therefore, a component that could target TBCM would be a potent candidate for TB drug development to ameliorate its pathogenesis.

### Supplementary Information

**Additional file 1: Fig. S1.** The absence of *tbcm* mRNA in B∆*tbcm*. **Fig. S2.** B∆*tbcm *enhances cytokine production in BMDMs. **Fig. S3.** Complementation of the ∆*tbcm* mutation in BCG. **Fig. S4.** Restored capacity to inhibit apoptosis by complementation of *tbcm* in a mouse model. **Fig. S5.** Bacterial CFU in BMDM was reduced at B∆*tbcm* and recovered by complementation of *tbcm. Fig. S6.* TOM20 expression level was recovered by complementation of ∆*tbcm *mutation. **Fig. S7.** Uncropped blot images of western blots shown in Fig. 3. **Fig. S8.** Uncropped blot images of western blots shown in Fig. 5. **Fig. S9.** Uncropped blot images of western blots shown in Fig. 6. **Table S1.** Used antibodies for western blot. **Table S2.** Primer sets for RT-qPCR.

**Additional file 2.** The raw RNA-sequencing data of infected BMDMs with wild-type BCG or BΔ*tbcm*.

## Data Availability

All data presented in the study are presented in the manuscript and Additional files 1 and 2. Further inquiries of datasets are available directed to the corresponding author.

## Abbreviations

TBCM: Tuberculosis chorismate mutase
B∆*tbcm*: BCG ∆*tbcm* mutant
BMDM: Bone marrow-derived macrophage
MOMP: Mitochondrial outer membrane permeabilization
C∆*tbcm*: Complementation of B∆*tbcm*
TB: Tuberculosis
BCG: *Mycobacterium bovis* BCG
Mtb: *Mycobacterium tuberculosis*
*nuoG*: NADH-quinone oxidoreductase subunit G
NADH-1: Type-1 NADH dehydrogenase
PtpA: Tyrosine phosphatase
Cpn60.2: Chaperonin60.2
CM: Chorismate mutase
DC: Dendritic cell
BMDC: Bone marrow dendritic cell
TLR-4: Toll-like receptor 4
OADC: Oleic albumin dextrose catalase
ADC: Albumin dextrose catalase
pBTdel: Plasmid used in construction of BCG *tbcm*
PCR: Polymerase chain reaction
Hsp65: Heat shock protein 65
EDTA: Ethylenediaminetetraacetic acid
SDS: Sodium dodecyl sulfate
gDNA: Genomic DNA
CFU: Colony forming unit
MOI: Multiplicity of infection
FBS: Fatal bovine serum
RPMI: Roswell park memorial institute
RT‒qPCR: Real-time quantitative PCR
TBS-T: Tris-buffered saline with tween 20
PBS: Phosphate-buffered saline
ECL: Enhanced chemiluminescence
RT: Room temperature
HRP: Horseradish peroxidase
TMRE: Tetramethylrhodamine, ethyl ester
JC-1: 5,5ʹ, 6,6ʹ-Tetrachloro-1,1ʹ, 3,3ʹ-tetraethylbenzimidazolylcarbocyanine, iodide
FCCP: Carbonylcyanide-p-trifluoromethoxyphenylhydrazone
mtROS: Mitochondrial reactive oxygen species
H&E: Hematoxylin and eosin
TUNEL: Terminal deoxynucleotidyl transferase dUTP nick end labeling
DAB: 3,3ʹ-Diaminobenzidine
ICC: Immunocytochemistry
PFA: Paraformaldehyde
BAX: Bcl-2 associated X protein
BAK: Bcl-2 homologous antagonist killer
BSA: Bovine serum albumin
DAPI: 4ʹ, 6-Diamidino-2-phenylindole
TNF-α: Tumor necrosis factor-α
IL-10: Interleukin-10
CD4: Cluster of differentiation 4
PI: Propidium iodide
Bcl-2: B-cell lymphoma 2
Apaf-1: Apoptotic protease activating factor-1
TRIM39: Tripartite motif containing 39
SARM1: Sterile alpha and Toll/IL-1 receptor motif containing 1
SLC25A26: Solute carrier family 25 member 26
NDUFA8: NADH dehydrogenase [ubiquinone] 1 alpha subcomplex subunit 8
DTYMK: Deoxythymidylate kinase
ΔΨ_m_: Mitochondrial membrane potential
TOM20: Translocase of the outer mitochondrial membrane 20
MTBC: *Mycobacterium tuberculosis* Complex
OCR: Oxygen consumption rate
