# Supplementary material for: A secreted form of chorismate mutase (Rv1885c) in Mycobacterium bovis BCG contributes to pathogenesis by inhibiting mitochondria-mediated apoptotic cell death of macrophages
Source: J Biomed Sci. 2023 Dec 18;30:95. doi: 10.1186/s12929-023-00988-2 (PMC10729386; doi:10.1186/s12929-023-00988-2)
Supplement: Supplementary file 1 — Additional file 1: Fig. S1. The absence of tbcm mRNA in B∆tbcm. Fig. S2. B∆tbcm enhances cytokine production in BMDMs. Fig. S3. Complementation of the ∆tbcm mutation in BCG. Fig. S4. Restored capacity to inhibit apoptosis by complementation of tbcm in a mouse model. Fig. S5. Bacterial CFU in BMDM was reduced at B∆tbcm and recovered by complementation of tbcm. Fig. S6. TOM20 expression level was recovered by complementation of ∆tbcm mutation. Fig. S7. Uncropped blot images of western blots shown in Fig. 3. Fig. S8. Uncropped blot images of western blots shown in Fig. 5. Fig. S9. Uncropped blot images of western blots shown in Fig. 6. Table S1. Used antibodies for western blot. Table S2. Primer sets for RT-qPCR. [file 12929_2023_988_MOESM1_ESM.docx]

**Additional file 1.**

**A secreted form of chorismate mutase (Rv1885c) in *Mycobacterium bovis* BCG contributes to pathogenesis by inhibiting mitochondria-mediated apoptotic cell death of macrophages**

Mi-Hyun Lee^1, 2, 7,^ Hye Lin Kim^1,5^, Hyejun Seo^1,5,6^, Sangkwon Jung^1,5^

and Bum-Joon Kim^1,2,3,4,5,6,7*^

This Word file includes:

Additional materials

Additional Fig. S1 to S9

Additional Table S1 and S2

**Additional materials**

**Genomic DNA extraction**

The colonies of mycobacteria incubated on 7H10 agar supplemented with 0.2% glycerol and 10% OADC for 2 to 4 weeks were used to extract the mycobacterial gDNA. For lysis, five loopfuls of cells were lysed in 200 µL of lysis buffer (15% sucrose, 0.05 M EDTA, 0.05 M Tris-Cl; pH 8.0; filtered and stored at room temperature) and briefly vortexed. Before reacting the samples at 37°C and 160 rpm overnight, lysozyme (Sigma, 10837059001) was added at a final concentration of 20 mg/ml. After incubation at 37°C, 6.25 µL of 20 mg/ml proteinase K (Meridian, bio37084), 100 µL of 20% SDS (Sigma, L3771), and 8 µL of RNaseA/T1 (Thermo, EN0551) were added and reacted for 10 min at 65°C.
 To purify gDNA, 1 volume of phenol‒chloroform-isoamyl alcohol (25:24:1; Invitrogen) was added and mixed well. The sample was centrifuged at 13,000 rpm for 10 min at 4°C, and the supernatant was collected. To precipitate and wash gDNA, 0.6 volumes of isopropanol were added, and the sample was stored at -20°C for 30 min, centrifuged, and washed with 70% ethanol. After the pellet was dried, it was redissolved in 20 µL of TE buffer (10 mM Tris-Cl, 1 mM EDTA; pH 8.0).

**Growth curve analysis**

To prepare stocks of wild-type BCG and B∆*tbcm*, colonies of each mycobacterium were seeded into complete 7H9 broth and grown to an OD_600_ of 1.0. Mycobacterial stocks were stored in a deep freezer (at −80°C). Each frozen stock was seeded in 200 ml of complete 7H9 broth at initial OD_600_ of 0.005. The OD value of the culture of wild-type BCG and B∆*tbcm* was measured every 2~3 days to generate a growth curve.

**Cell culture and BMDM differentiation**

BMDMs were generated from the femurs and tibias of 8- to 12-week-old BALB/c mice (female). Bone marrow cells were harvested by flushing the femurs and tibias. The collected cells were resuspended in red blood cell lysis buffer (Merck, R7757) and filtered through a 70 µM cell strainer (SPL, 93070). The cells were resuspended in RPMI 1640 medium containing 10% FBS (Gibco, 12483020), 1% penicillin‒streptomycin (Welgene, LS202-02), 30 ng/ml M-CSF (Peprotech, 315-02) and 1% L-glutamine (Sigma, G7513). The cell suspension was cultured in a 100 mm cell culture dish (SPL, 20100) and incubated at 37°C and 5% CO_2_ for 7 days. Cells of the murine macrophage line J774A.1 (American Type Culture Collection, ATCC TIB-67) were cultured in complete RPMI 1640 and incubated at 37°C and 5% CO_2_.

**Histology**

The collected lung tissues were fixed in 4% paraformaldehyde (SolMate, SM-P01-100) and embedded into paraffin blocks. Sections (4-5 μm thick) were prepared and deparaffinized with a xylene/ethanol solution. Rehydrated specimens were stained with hematoxylin and eosin (H&E) to evaluate the level of inflammation caused by infection. To detect apoptotic cells in the lung tissue, a TUNEL Assay Kit - HRP-DAB (Abcam, ab206386) was used according to the manufacturer’s protocol, and stained tissues were observed using a light microscope.

**Cytokine assay**

The splenocytes from infected mice were seeded at 1x10^6^ cells/well in a 96-well round bottom plate (SPL, 34096) and incubated at 37°C and 5% CO_2_. After 3 days, supernatants were collected. In the case of infected BMDMs (1x10^6^), supernatants were collected 24 and 48 h post infection. To measure the levels of cytokines such as TNF-$\alpha$ (Invitrogen, 88-7324-88) and IL-10 (Invitrogen, 88-7105-88), ELISA kits were used.

**CFU assay**

The intracellular CFU assay was performed at 24 and 48 h post infection. After treating BMDMs and J774A.1 cells (5x10^5^) with PBS containing 1% Triton X-100, the cells were serially diluted with PBS. In the case of organs, three organs (spleen, lung, liver) were homogenized with a 70 µM cell strainer (SPL, 93070) and diluted with PBS. The diluted solution was plated and cultured on 7H10 agar plates containing glycerol and OADC for 3 weeks at 37°C and 5% CO_2._

**Library preparation and sequencing**

We request RNA-sequencing and its analysis to e-biogen (Seoul, Korea). For library preparation of control and test RNAs, a QuantSeq 3’ mRNA-Seq Library Prep Kit (Lexogen, Inc., Austria) was used by following the manufacturer’s instructions. Each RNA sample was prepared with a primer containing an Illumina-compatible sequence. Reverse transcription was processed after 5’ end of primer was hybridized to the RNA. Second strand synthesis was instigated by a random primer with an Illumina-compatible linker sequence of its 5’ end. The double-stranded library underwent purification by magnetic beads to eliminate all reaction components. Furthermore, the library was amplified to incorporate complete adapter sequences for cluster generation. The final library was purified from PCR components and high-throughput sequencing was carried out as single-end 75 sequencing using NextSeq 550 (Illumina, Inc., USA).

**RNA isolation and quantitative real-time PCR**

Total RNA was isolated from infected BMDMs and J774A.1 cells (5x10^5^) at 0, 2, 4, 12 and 24 h post infection using TRIzol reagent (Invitrogen, 15596018). The RNA isolated from wild-type BCG and B∆*tbcm* was also prepared using TRIzol reagent. cDNA was synthesized using a SensiFAST^TM^ cDNA Synthesis Kit (Bioline, BIO-65054) according to the manufacturer’s protocol. RT-qPCR was performed using a SensiFAST^TM^ SYBR® Lo-ROX Kit (Bioline, BI0-94020). The relative expression level was calculated using the housekeeping gene β-actin for cells or the *hsp65* for mycobacteria as a control. The primer sets used in RT‒qPCR are shown in the Table S2.

**Cytochrome c ELISA**

Infected BMDMs and J774A.1 cells (5x10^5^) were suspended in PBS containing 0.5% Triton X-100 at 8 h post infection. Cytochrome c release was evaluated using Rat/Mouse Cytochrome c Quantikine ELISA Kit (R&D Systems, MTCT0) according to the manufacturer’s protocol.


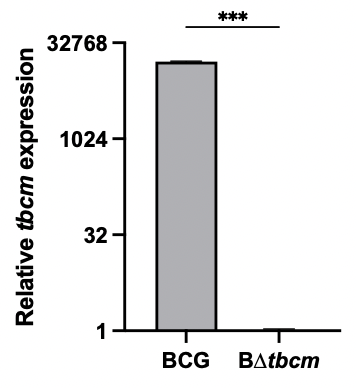


**Fig. S1. The absence of *tbcm* mRNA in B∆*tbcm***

The relative expression levels of *tbcm* mRNA for both BCG and B∆*tbcm* were compared by normalizing them to the expression levels of *hsp65* using RT-qPCR.

**
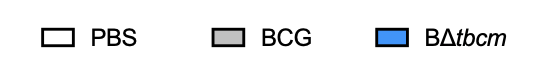
**
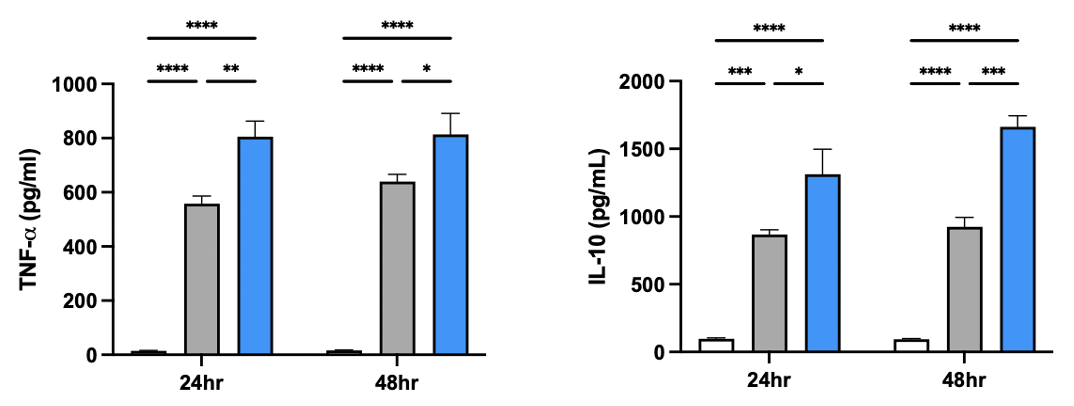


**Fig. S2. B∆*tbcm* enhances cytokine production in BMDMs**

BMDM were infected with BCG or B∆*tbcm* at 10 M.O.I for 24 h or 48 h. TNF-$\alpha$ (left) and IL-10 (right) were detected in the culture supernatants by ELISA.

**
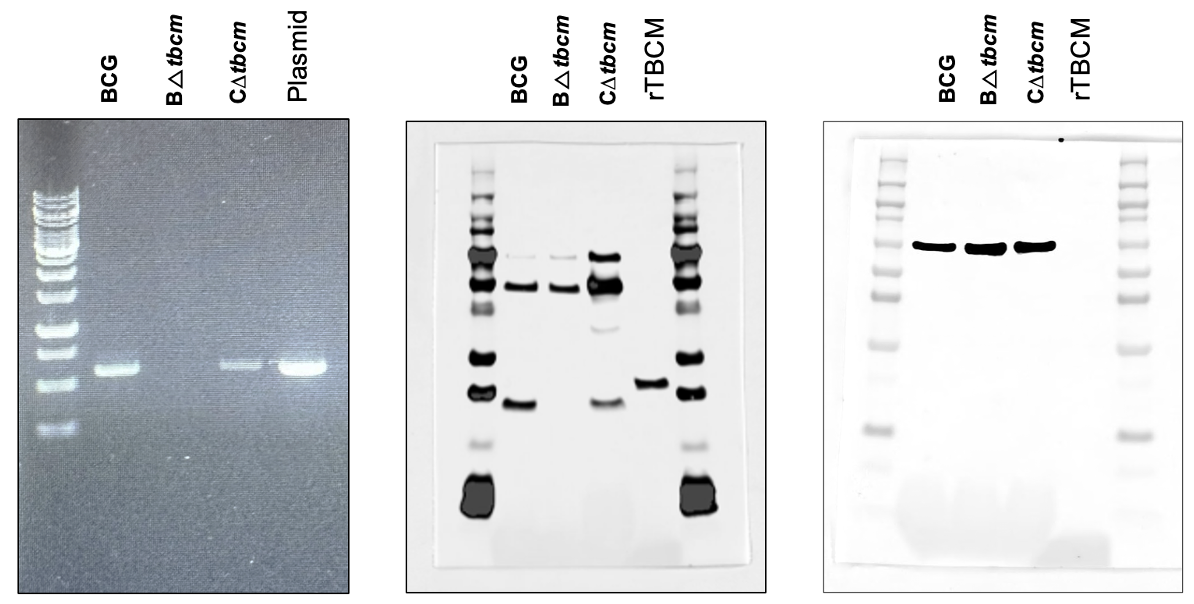
**

**Fig. S3. Complementation of the ∆*tbcm* mutation in BCG.**

Whole gel image of PBCR confirmation of *tbcm* gene (600bp) expression in C∆*tbcm* (left). Uncropped blot image of TBCM protein expression in C∆*tbcm* (middle) and hsp65 protein expression for normalization (right).

C

**
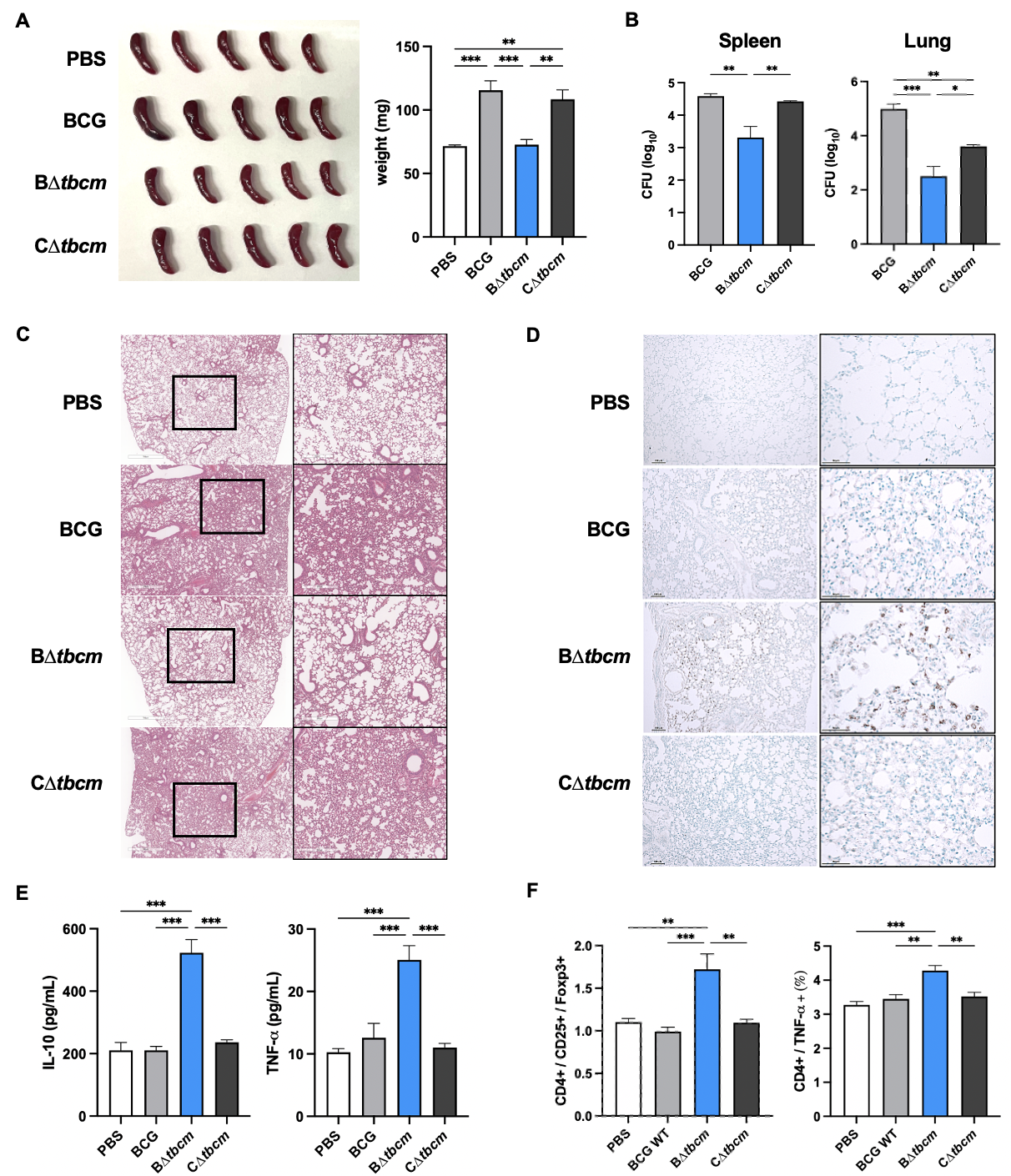
**

**Fig. S4. Restored capacity to inhibit apoptosis by complementation of *tbcm* in a mouse model.**

Female C57BL/6 mice (n=5 per group) were infected with BCG, B∆*tbcm*, or C∆*tbcm* (1x10^6^ CFU) intravenously and sacrificed after 4 weeks. (A) Comparison of spleen size (left) and weight (right). (B) The bacterial burdens in spleens and lungs were measured by the CFU assay. (C) Histopathological images (H&E staining) of lung tissues (scale bars, 300 $\mu$m or 700 $\mu$m). (D) Apoptotic cells in lung tissues were detected by the TUNEL assay (scale bars, 50 $\mu$m or 100 $\mu$m). (E) Splenocytes were isolated from infected mice and incubated for 3 days. The levels of IL-10 (left) and TNF-$\alpha$ (right) were detected in the culture supernatants of splenocytes by ELISA. (F) CD25+/Foxp3+ regulatory T cell and CD4+/ TNF-α+ T cell subsets were analyzed in splenocytes by flow cytometry. One-way ANOVA with Tukey’s multiple comparison test. (*P<0.05, **P<0.01, ***P<0.001). Abbreviations: BCG, wild-type BCG; BΔ*tbcm*, BCG Δt*bcm* mutant; CΔ*tbcm,* complemented mutant.

**
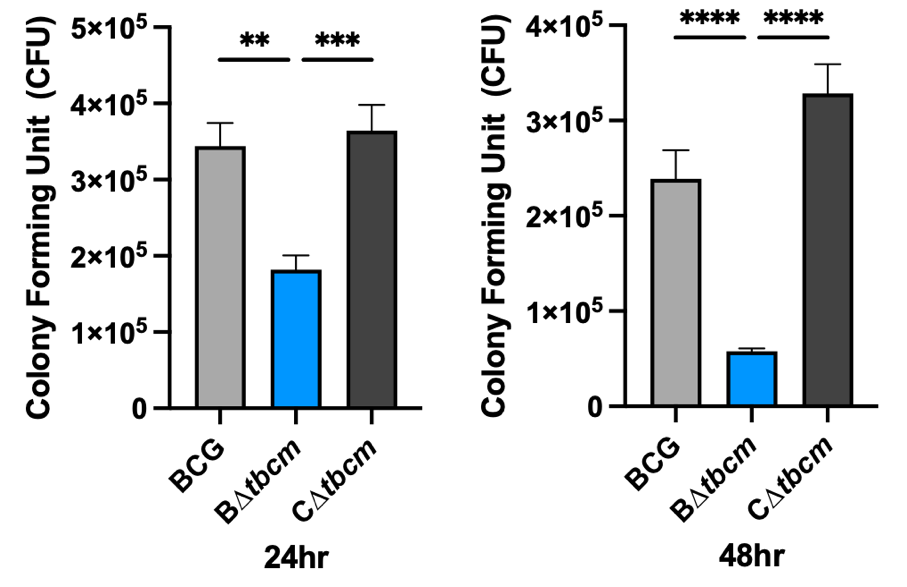

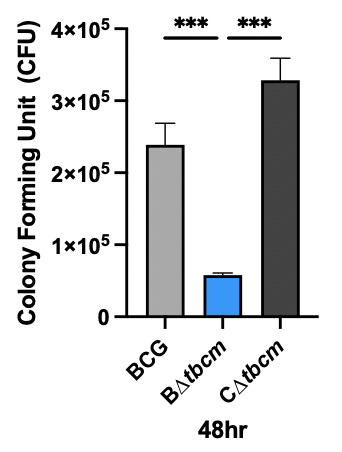
**

**Fig. S5. Bacterial CFU in BMDM was reduced at B∆*tbcm* and recovered by complementation of *tbcm***

BMDMs (BALB/c, female) were infected with BCG, B∆*tbcm*, or C∆*tbcm* for 24h or 48h.

**
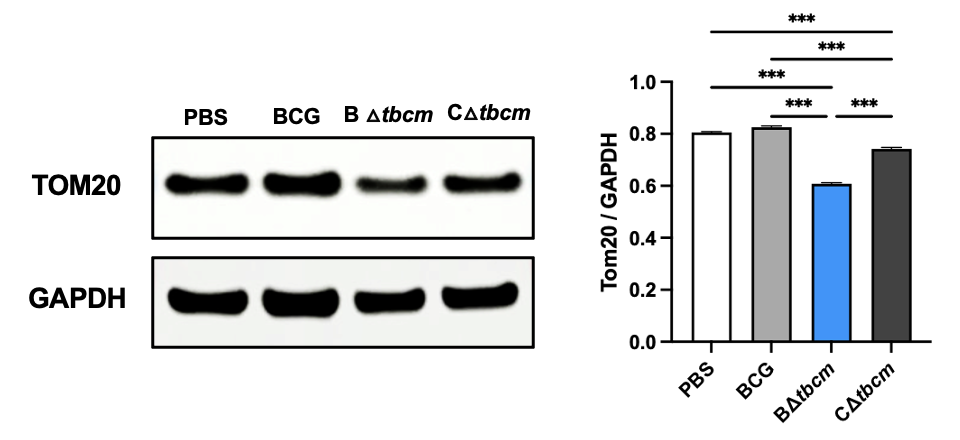
**

**Fig. S6. TOM20 expression level was recovered by complementation of ∆*tbcm* mutation.**

J774A.1 cells were infected with each strain for 18h. Immunoblot analysis of infected macrophages (whole cell lysate).


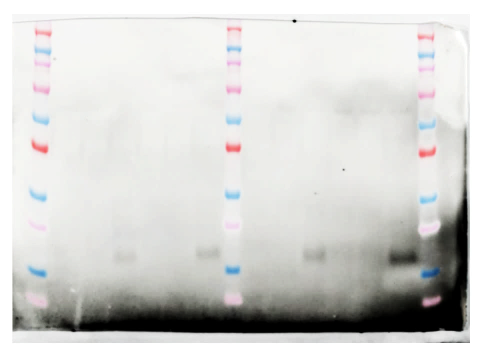


15kDa

20kDa


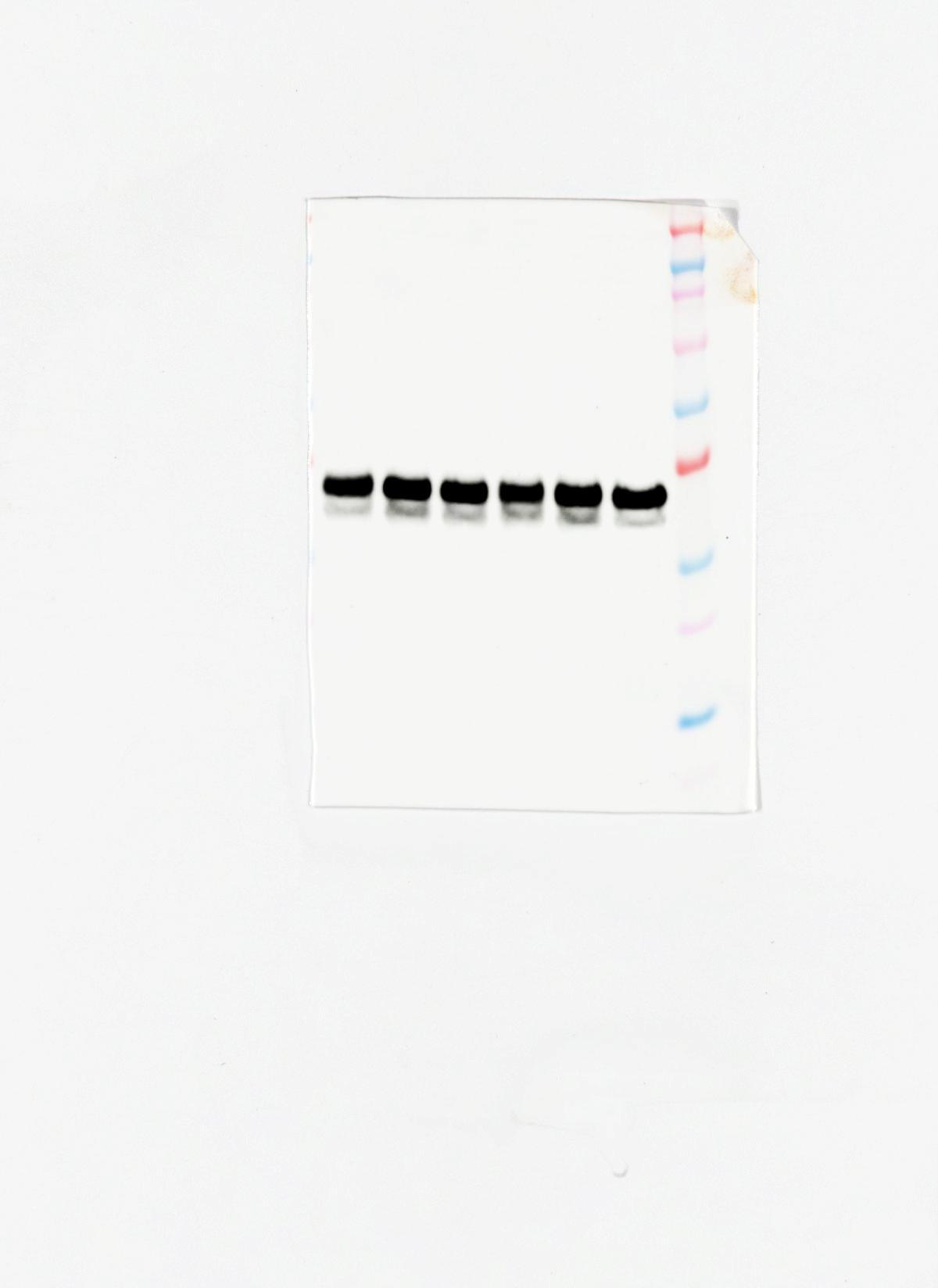


30kDa

40kDa

**Fig. S7. Uncropped blot images of western blots shown in Fig. 3**

Cleaved-caspase-3 (left), GAPDH (right)


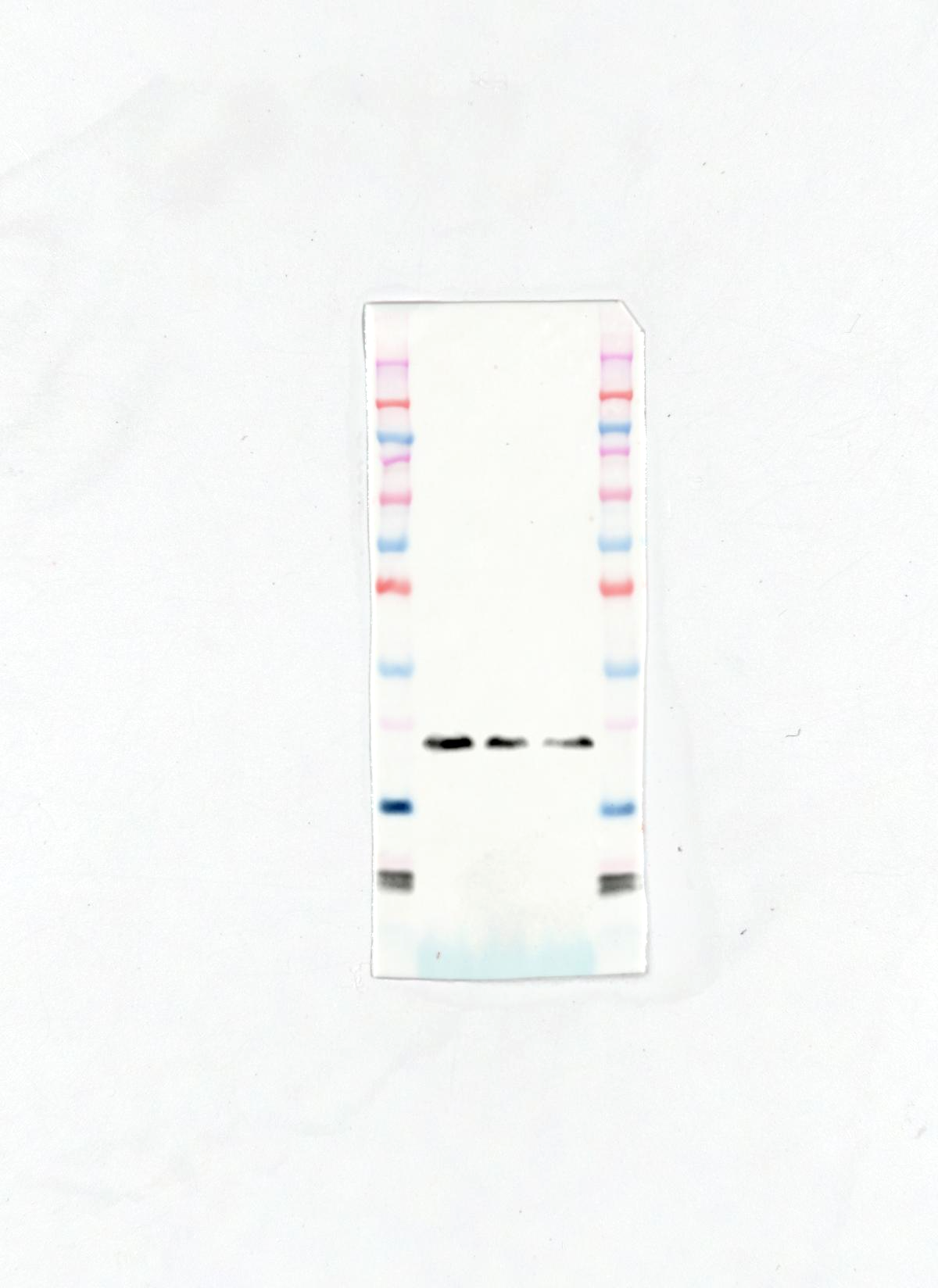


15kDa

20kDa


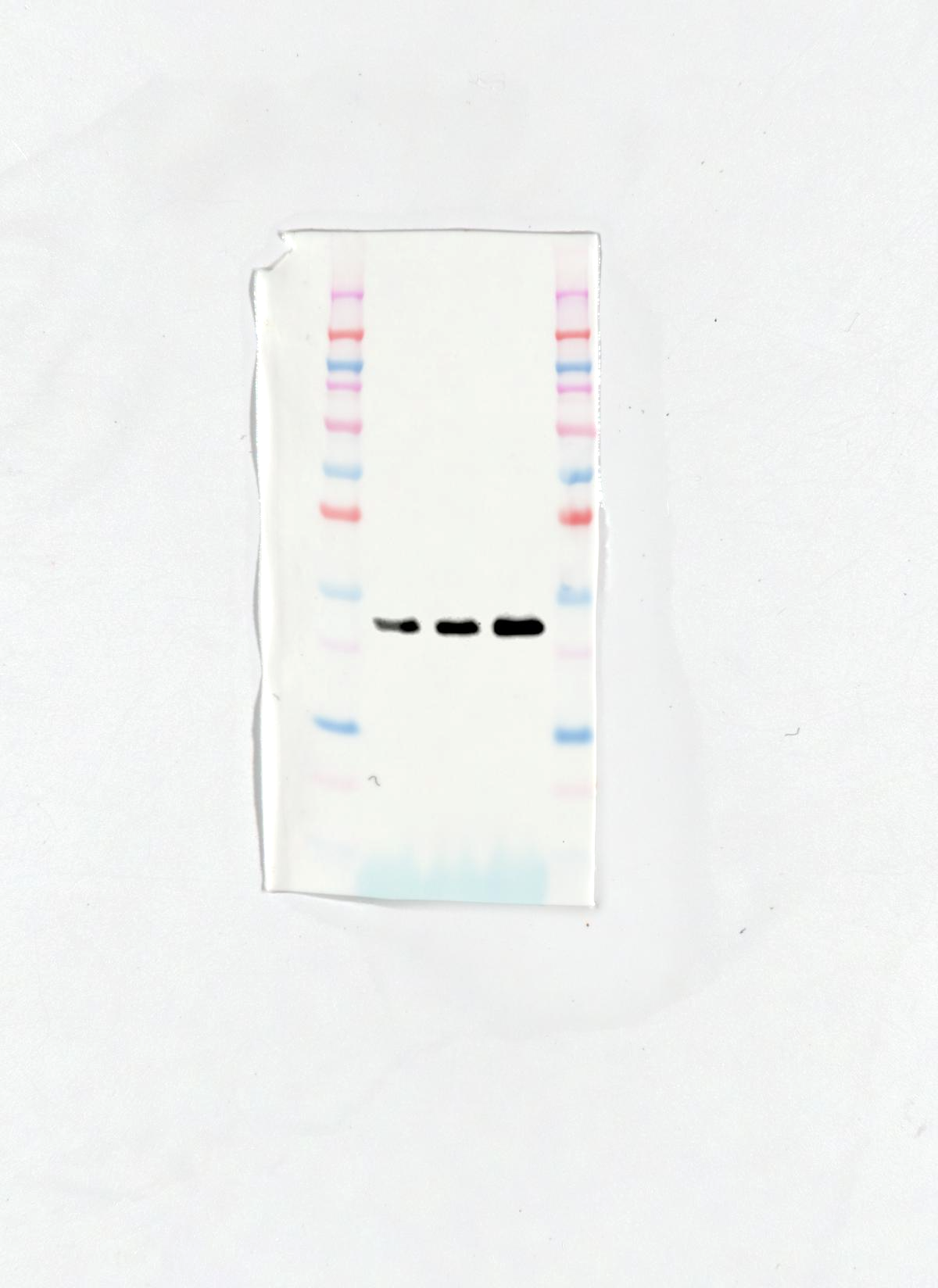


20kDa

30kDa

A

B


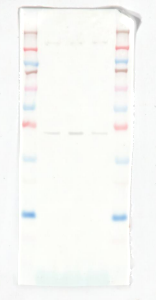

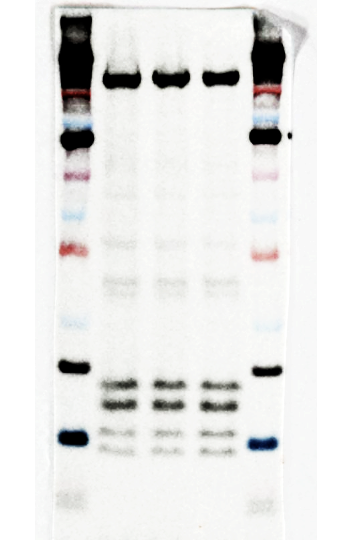


15kDa

20kDa

30kDa

40kDa

C

D


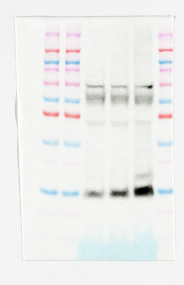


10kDa

15kDa

E


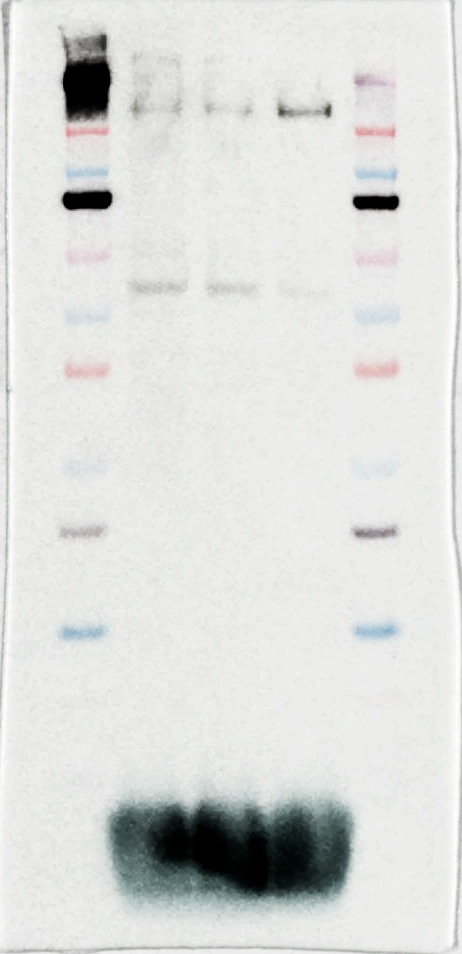


F

15kDa

20kDa


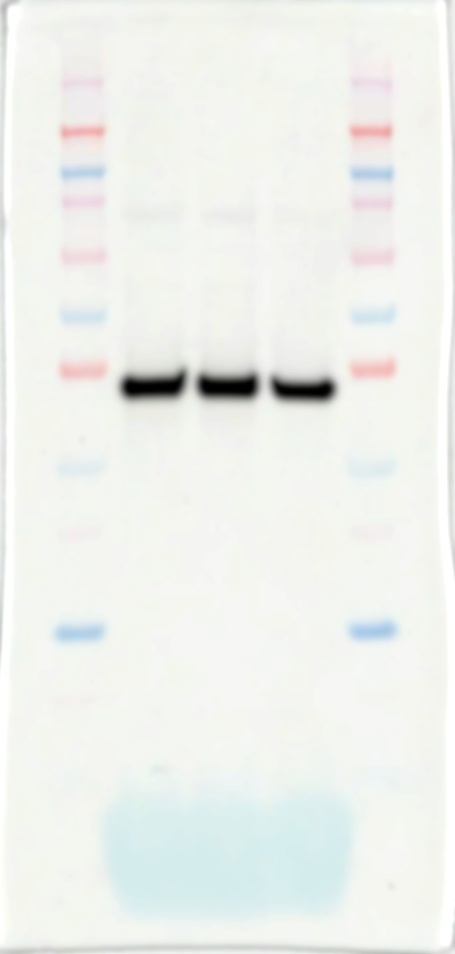


30kDa

40kDa

G


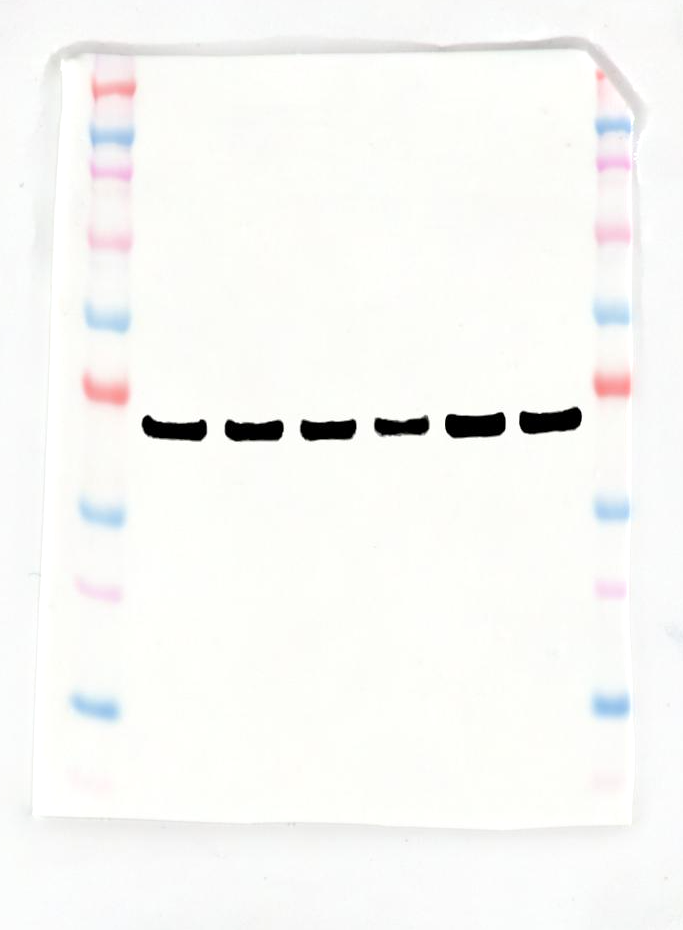


30kDa

40kDa

I


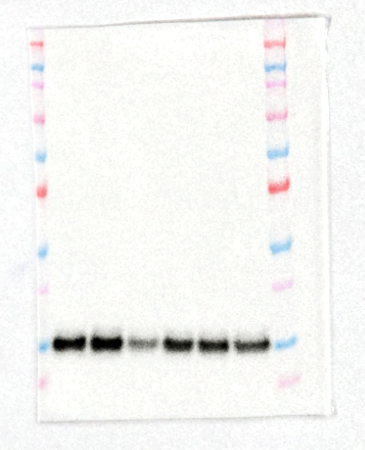


15kDa

20kDa

H

**Fig. S8. Uncropped blot images of western blots shown in Fig. 5**

Mitochondrial fraction **A)** BAX, **B)** BAK, **C)** COX IV and **D)** GAPDH

Cytosolic protein **E)** Cytochrome c**, F)** COX IV and **G)** GAPDH

Whole cell lysate **H)** TOM20 and I**)** GAPDH

B

A


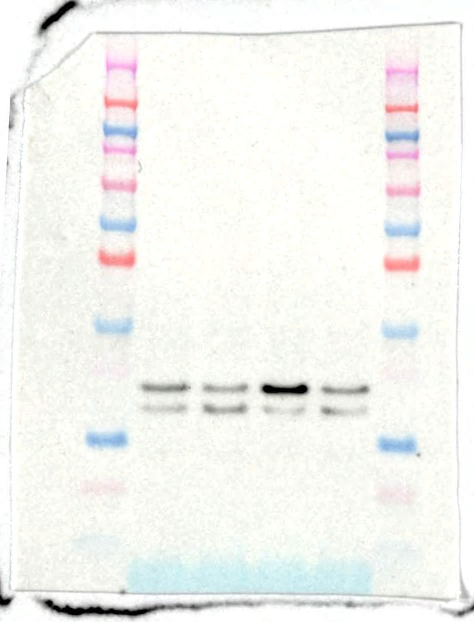


15kDa

20kDa

A


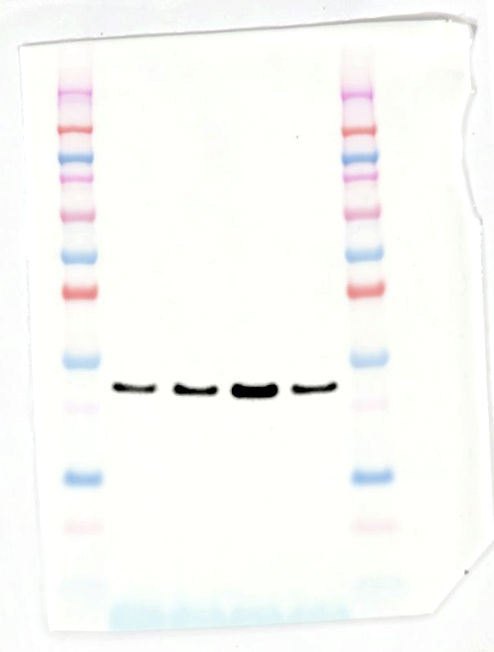


20kDa

30kDa

B


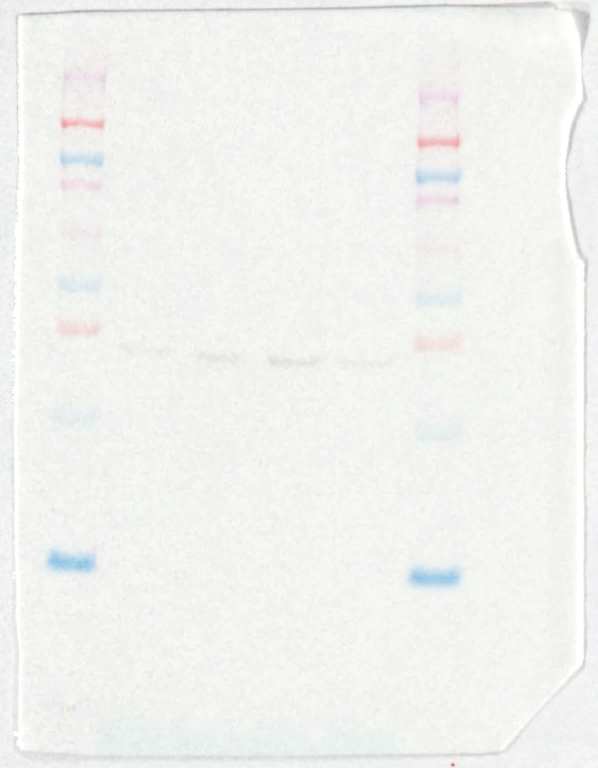


30kDa

40kDa


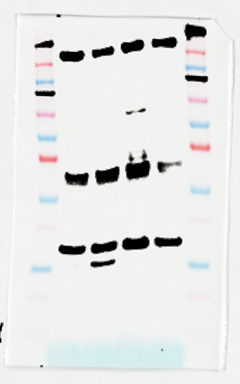


15kDa

20kDa

C

D

C

D


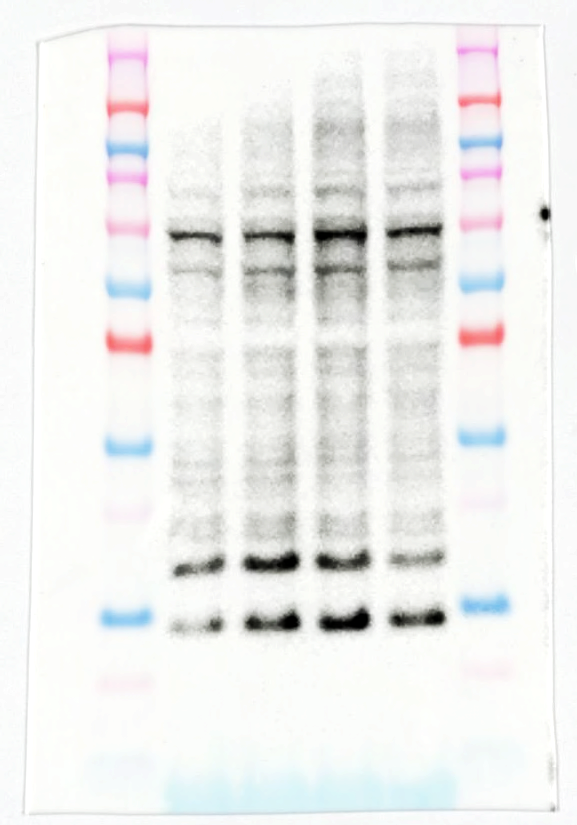

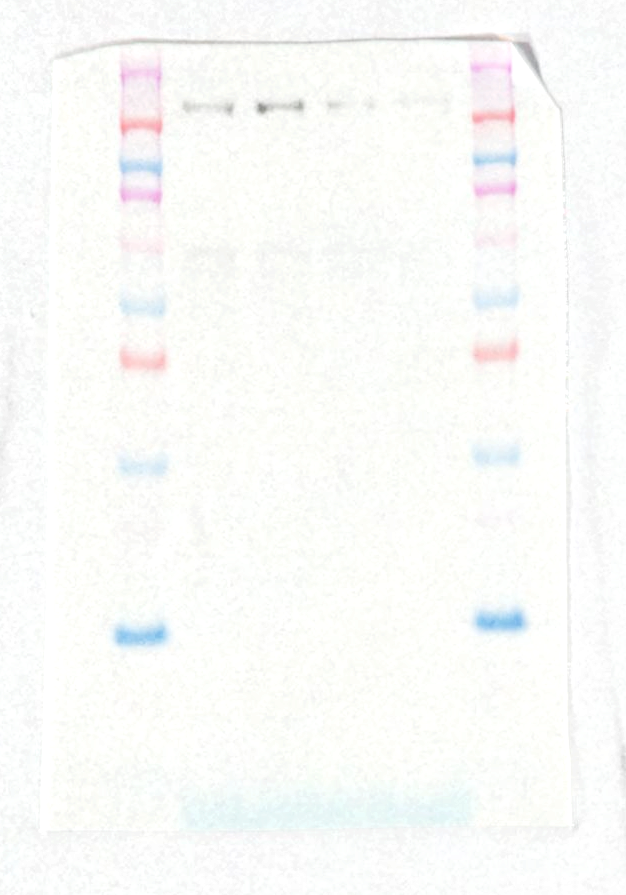

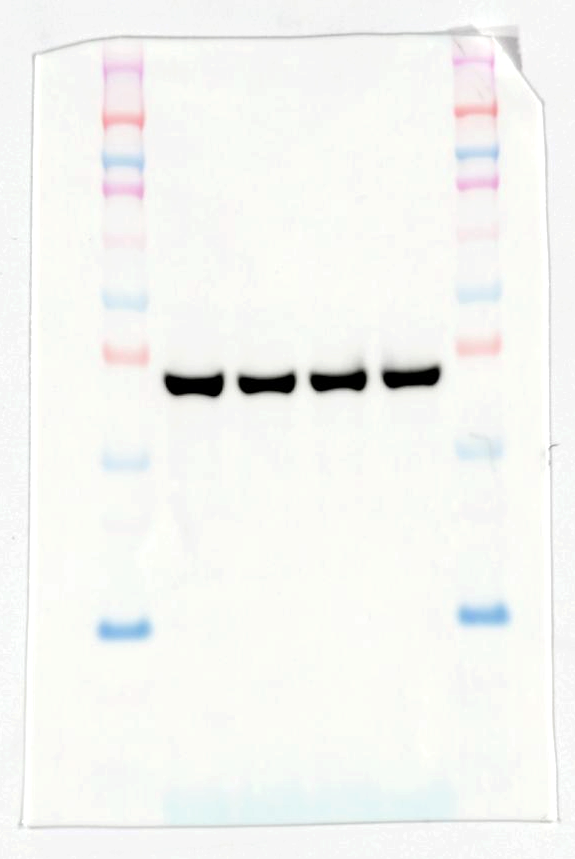


10kDa

15kDa

30kDa

40kDa

E

F

G

15kDa

20kDa

**Fig. S9. Uncropped blot images of western blots shown in Fig. 6**

Mitochondrial fraction **A)** BAX, **B)** BAK, **C)** COX IV and **D)** GAPDH

Cytosolic protein **E)** Cytochrome c**, F)** COX IV and **G)** GAPDH

| **Antibody** | **Dilution** | **Company** | **Catalog number** |
| --- | --- | --- | --- |
| TBCM | 1:1000 | Bionics | - |
| Hsp65 | 1:200 | Santacruz | sc-57842 |
| Cleaved caspase-3 | 1:1000 | Cell signaling | 9661S |
| Tom20 | 1:1000 | Cell signaling | 42406S |
| Cytochrome c | 1:1000 | Cell signaling | 11940S |
| BAX | 1:1000 | Cell signaling | 2772S |
| BAK | 1:1000 | Cell signaling | 12105S |
| COXIV | 1:1000 | Cell signaling | 4850S |
| GAPDH | 1:1000 | Thermo fisher | MA5-15738-HRP |

**Table S1. Used antibodies for western blot**

Production of TBCM antibody was conducted by Bionics (Seoul, Korea).

| **Gene**  **symbol** | **Forward primer (5’-3’)** | **Reverse primer (5’-3’)** |
| --- | --- | --- |
| Bcl-2 | GAG TAC CTG AAC CGG CAT CT | TTG TTT GGG GCA GGT TTG TC |
| Caspase-7 | CCG AGT GCC CAC TTA TCT GT | ACC TGT CGC TTT GTC GAA GT |
| Caspase-9 | TTC CCA GGT TTT GTC TCC TG | GGG ACT GCA GGT CTT CAG AG |
| Apaf1 | GAT CCA CAC AGG CCA TCA CA | GGC GGG AGT CTA TGT TCC AC |
| Trim39 | TGG AGG TGA CTT CAG TAT CCA T | TCA CAT CCG CAA TTA GCT GTT |
| Sarm1 | TCG CAA TTT TGT CCT GGT G | AGC TTA AAG CAG TCA CAA TCT CC |
| Slc25a26 | TCT GGG GCA ACA GTG TGT AG | TAC TAA GTG TGT GCG GCG GT |
| Ndufa8 | GAG TTT ATG CTG TGC CGC TG | TAC TCT GTG AAA GGC TCC GC |
| Dtymk | GTC CTG TTC CTC CAG TTA C | AGC ATC CAC CAT CTT CCA |
| β-actin | GCA ATT ATT CCC CAT GAA CG | GGC CTC ACT AAA CCA TCC AA |
| tbcm | GCA AAG TTG GGC GAA GAT G | ATC CGA TTA TTC AGG GAG TCG ATC |
| hsp65 | GTC GAG GAG TCC AAC ACC TT | GAG CTG ACC AGC AGG ATG TA |

**Table S2. Primer sets for RT-qPCR**
